# Solution-Processed Inorganic Thermoelectric Materials: Opportunities and Challenges∇

**DOI:** 10.1021/acs.chemmater.2c01967

**Published:** 2022-09-21

**Authors:** Christine Fiedler, Tobias Kleinhanns, Maria Garcia, Seungho Lee, Mariano Calcabrini, Maria Ibáñez

**Affiliations:** † Institute of Science and Technology Austria (ISTA), Am Campus 1, 3400 Klosterneuburg, Austria

## Abstract

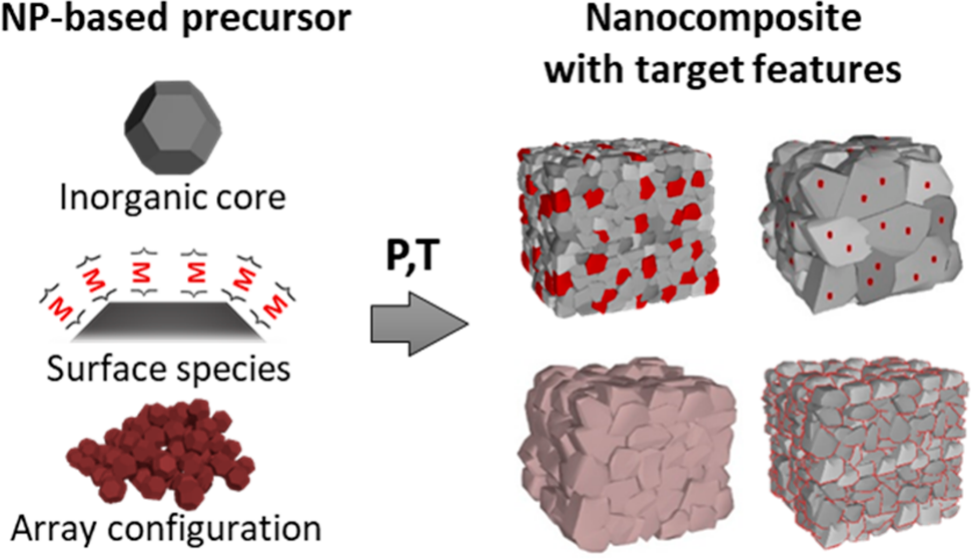

Thermoelectric technology requires synthesizing complex materials where not only the crystal structure but also other structural features such as defects, grain size and orientation, and interfaces must be controlled. To date, conventional solid-state techniques are unable to provide this level of control. Herein, we present a synthetic approach in which dense inorganic thermoelectric materials are produced by the consolidation of well-defined nanoparticle powders. The idea is that controlling the characteristics of the powder allows the chemical transformations that take place during consolidation to be guided, ultimately yielding inorganic solids with targeted features. Different from conventional methods, syntheses in solution can produce particles with unprecedented control over their size, shape, crystal structure, composition, and surface chemistry. However, to date, most works have focused only on the low-cost benefits of this strategy. In this perspective, we first cover the opportunities that solution processing of the powder offers, emphasizing the potential structural features that can be controlled by precisely engineering the inorganic core of the particle, the surface, and the organization of the particles before consolidation. We then discuss the challenges of this synthetic approach and more practical matters related to solution processing. Finally, we suggest some good practices for adequate knowledge transfer and improving reproducibility among different laboratories.

## 1 Introduction

Over 100 years ago, the direct and reversible conversion between heat and electricity was identified.^1^ This phenomenon, known as thermoelectricity, offers a sustainable path to produce electricity from waste heat. The potential of thermoelectric devices as energy harvesters can be envisioned from two different, yet both very important, perspectives. On one hand, these devices can be used to improve the overall energy utilization. The energy flow of most developed countries indicates that approximately 60% of the energy produced is wasted,^2^ mostly as heat; therefore, partially recovering the waste heat is a clear strategy to reduce our primary energy production. On the other hand, thermoelectric devices can be used in low-power devices, especially those that require autonomy, such as sensors and transmitters in remote or difficult-to-access locations that are crucial for the development of the “Internet of Things” (IoT).^3^ In this regard, thermoelectric devices are an ideal choice, as temperature differences are ubiquitous and the devices are robust, maintenance-free, scalable, and compact.

Furthermore, the reversible nature of thermoelectric devices allows them to be operated as precise coolers for small-scale temperature control.^4^ Such localized cooling is crucial in infrared detectors, microelectronics, and optoelectronics, among others, where space is limited and heat dissipation is localized.^5^ Another benefit of thermoelectric devices for heating and cooling applications is that they allow moving away from centralized thermal management toward distributed thermal management, where only the necessary area is heated or cooled.^6^

Despite the impactful prospects of thermoelectric devices, to date their implementation has been restricted to niche applications where there is no alternative option, and neither cost nor efficiency is the most relevant factor. One clear example is radioisotope thermoelectric generators used in space exploration.^7^ So far, the large-scale implementation of thermoelectric devices has been hindered by high costs, which come from pricey raw materials, energy-intensive processing methods, and the low efficiency of these devices. These issues must be addressed for thermoelectric devices to be widespread and contribute to improving our energy consumption.

The efficiency of a thermoelectric device is directly linked to the properties of the materials used as thermoelectrics. To maximize material performance, a high electrical conductivity (σ), a large Seebeck coefficient(*S*), and a low thermal conductivity (κ) are necessary. These properties are grouped into the dimensionless material figure of merit (*zT*) defined as *zT* = *σS*^2^*Tκ*^–1^, where *T* is the absolute temperature. One focus of research in thermoelectrics is seeking new material systems with the highest possible *zT* that are nontoxic, inexpensive, and sustainable, so they can be used for mass production. This goal has been pursued since Ioffe, in 1949, identified that maximizing the average *zT* of the material maximizes the performance of thermoelectric devices.^8^ However, the problem is that these three macroscopically measurable transport parameters (*σ*, *S*, and *κ*) are strongly related; they depend on a series of material electronic, vibrational, and structural parameters that are unfavorably interconnected.^9^ Semiconductors are thus the most efficient materials, where this delicate balance between heat and charge transport is best controlled.

**Figure 1:**
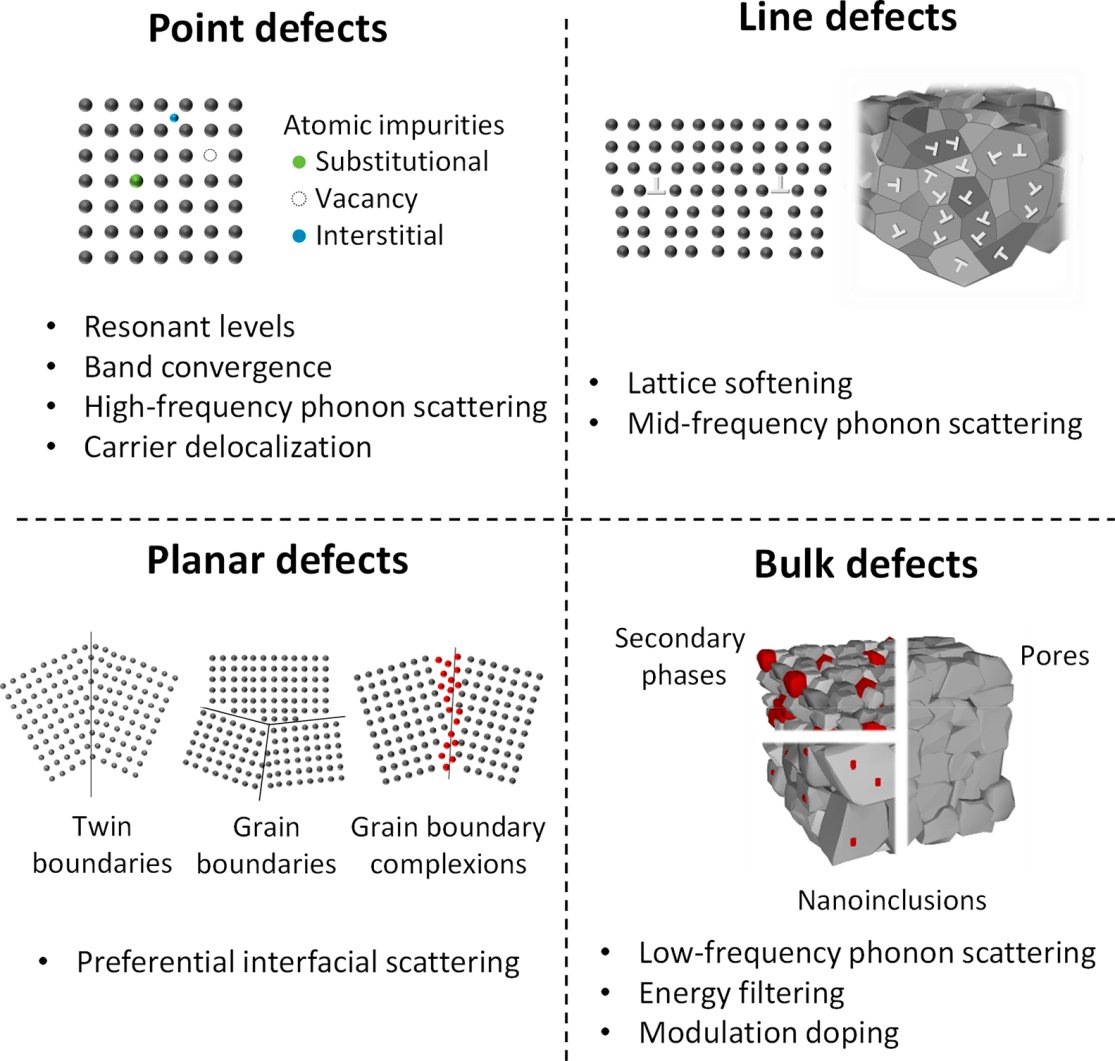
Multiscale defect engineering strategies to enhance the thermoelectric material figure of merit. Different strategies to improve the material performance rely on introducing and controlling different kinds of defects at different length scales (atomic to micro). Here, we present the most relevant ones that have allowed the development of record-performing thermoelectric materials for different material families.

Strategies to maximize *zT* are based on innovative transport mechanisms that alter the adversely dependent transport properties, allowing them to be tuned more independently. Examples include resonant levels,^10^ band convergence,^11^ modulation doping,^12^ nanostructuring,^13^ lattice softening,^14,15^ Anderson-like localization,^16^ and interfacial preferential scattering.^17^ These mechanisms are enabled by defects at different length scales, such as point defects (0D), linear defects (1D), planar defects (2D), and bulk defects (3D) (Figure 1). Therefore, the best-performing thermoelectric materials have complex microstructures, where both the structural and chemical nature of the multiscale defect ensemble determine the interaction with charge carriers and phonons.^18^ This indicates that the way to achieve record-performing materials is to develop materials beyond unit cells, harnessing functionality from what would be normally considered imperfections.

Point defects, i.e., doping and alloying, can be well-controlled using conventional methods. However, carefully curating line, planar, and bulk defects within polycrystalline materials creates challenges in chemistry. Such challenges should be addressed with innovative synthetic methods that provide unique opportunities to control and tune defect formation in semiconductors, coupled with a thorough characterization for accurate correlations.

In this perspective, we propose a synthetic strategy that uses well-defined powders to direct the chemical transformation into dense inorganic thermoelectric materials with targeted features. The best way to achieve superb control of the powder properties is through solution processing.^19,20^ With this in mind, we first cover the opportunities of synthesizing the powder in solution, followed by the challenges that must be addressed to execute this synthetic idea successfully. Then, we propose good practices for reporting the synthesis of solution-processed thermoelectric materials to improve reproducibility among different laboratories. Finally, we present our outlook on how solution-processed materials could enable the *synthesis by design* of inorganic semiconductors, impacting not only the thermoelectric field but also others.

## 2 The Opportunities

Common thermoelectric materials are dense polycrystalline inorganic semiconductors typically prepared in two steps: the semiconductor is first synthesized in powder form, then consolidated into a dense sample,^21^ usually shaped in the form of a disk or a bar. In order to provide the consolidated material with a density as close as possible to the theoretical one, pressure-assisted sintering techniques are preferred, such as hot pressing or spark plasma sintering. In these techniques, uniaxial pressure is usually applied while the sample is heated under a vacuum or an inert gas. The transformations that occur during consolidation define the microstructure of the material (i.e., defect types, density, and distribution), which directly affects its electronic, thermal, and mechanical properties. Such transformations are controlled by both the consolidation conditions (reaction conditions, including temperature, pressure, atmosphere, etc.) and the powder properties.

In this Perspective, we focus on the possibility of designing powders using solution processing methods to achieve bulk materials with specific features. However, we do not comment on the important role of the consolidation technique and reaction conditions in the formation of the solids.

Characteristics of the powder, such as particle size and shape, composition, and surface chemistry, determine densification, grain growth, side reactions, and defect formation, overall controlling the material’s final microstructure.^22^ Powders are commonly prepared by high-energy milling;^23,24^ however, these processes do not allow a precise adjustment of the particle properties. Therefore, an opportunity to control the material microstructure is lost. This limitation can be overcome by synthesizing the powders in solution.

**Figure 2:**
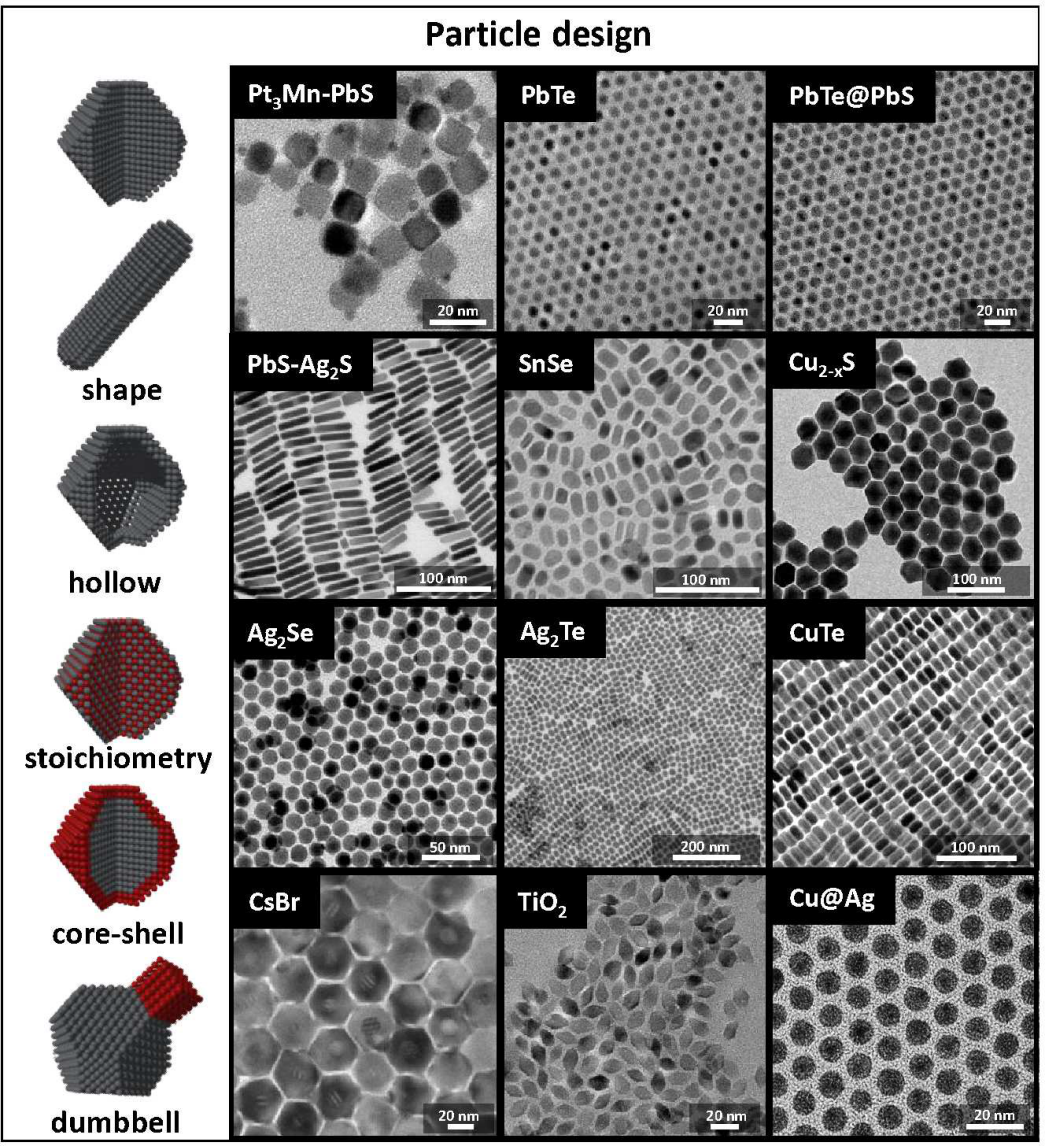
Examples of the versatility in NP architecture. In colloidal synthesis, ligands are used to dissolve the precursors, control nucleation and growth, and finally provide colloidal stability to the NPs, leading to much higher quality and versatility than surfactant-free hydro and solvothermal methods. NPs of different shapes (spheres, rods, and dumbbells), sizes (1–100 nm), and compositions (metals, oxides, and semiconductors) can be prepared by carefully choosing the reactants, ligands, and reaction conditions. Examples of different possible designs (left) and examples of solution-processed NPs with various sizes and morphologies. Reproduced with permission from ref (42). Copyright 2016, from the Royal Society of Chemistry. Reproduced from refs (43) and (44). Copyright 2019 and 2017, respectively, American Chemical Society.

Solution-based syntheses offer the possibility of producing nanoparticles (NPs) with carefully curated features compared to those produced by mechanical methods. Thermoelectric materials have been prepared using aqueous and solvothermal methods with low costs and high energy efficiencies.^25−27^ Yet, these methods do not provide a high level of control over the particle properties. Among the solution methods, surfactant-assisted synthesis, also known as colloidal synthesis,^28^ has outperformed any other known method, producing inorganic NPs with precise compositions and morphologies;^29,30^ therefore, it is the most promising method for precisely designing NP-based precursors. Colloidal syntheses use surfactants to dissolve the precursors, direct the synthesis, and provide colloidal stability,^31,32^ enabling the production of particles with an ever-increasing number of elements,^33^ the creation of nanoheterostructures with high sophistication levels,^34,35^ and extraordinary control over size, monodispersity, shape, crystal phase, and even surface termination (Figure 2).^36−44^

Despite the high level of control that can be achieved with colloidal synthesis, this strategy has not been intensively used to produce thermoelectric materials.^42^ Producing semiconductor powders using colloidal synthesis offers unique possibilities in materials’ design and holds enormous potential for the next generation of complex thermoelectric materials.

### 2.1 Precise Design of Nanoparticles for Solids with Targeted Defects

In the same way that metal complexes are converted into well-defined NPs in colloidal synthesis,^45,46^ NPs can be used as tunable precursors capable of evolving into bulk materials with specific structural features under proper reaction conditions. One might argue that using carefully curated NPs as precursors to produce dense solids is a futile effort, as those perfect aesthetic NPs will be “destroyed”. However, this can be looked at from another perspective in which the precise design of the NP-based precursor, i.e., the powder, gives us control over the processes that occur during the consolidation. In the approach proposed here, the particles would undergo not only sintering but also other transformations, including the decomposition of surface species,^47,48^ solid-state reactions,^49^ melting,^50^ etc., that would ultimately modify the structure and composition and hence the transport properties of the consolidated material. Moreover, it is important to bear in mind that although colloidal synthesis provides extraordinary control over the features of the NP (size, shape, crystal structure, etc.), not all of them need to be controlled at once to achieve the desired architectural features in the solid.

To discuss the possibilities that colloidal synthesis offers for tuning the structures of thermoelectric materials, we describe the NPs as consisting of an inorganic core (IC), generally but not exclusively crystalline, and surface species (surfactants or adsorbates).

#### 2.1.1 Inorganic Core Design

The most prominent characteristics of NPs, namely, the size, shape, crystallinity, and crystal phase, are defined by the IC. Therefore, precise control over such properties provides unique possibilities to tune the transformation of NPs into macroscopic solids.

##### 2.1.1.1 Size and Shape

One of the most notorious properties that affects the transport properties of thermoelectric materials is the size of the crystal domains.^51^ Hence, tuning the size and morphology of the precursors’ ICs is highly important to optimize the performance of the thermoelectric material. Moreover, ICs with different morphologies and sizes display different distributions of exposed crystallographic facets.^52^ Surface facets vary in their atomic arrangement, stoichiometry, free energy, and coordination environment.^53^ For instance, quasi-spherical CdSe ICs are known to possess {100} and {111} facets, whereas the surface of CdSe nanoplatelets is dominated by {100} facets.^54,55^ Together, these properties of the IC are crucial to control the reactivity during consolidation. Additionally, the size and shape of the IC change the ratio and type of surface termination atoms.^56,57^ These changes in stoichiometry at the IC level can be used to determine the final composition and defects, such as vacancies.^58^ Finally, anisotropic ICs (rods, platelets, or disks) can be used as precursors for macroscopic solids with a crystallographic texture.^42,59−63^

##### 2.1.1.2 “Nonequilibrium” Compositions and Phases

One of the most exciting avenues to explore is the use of NPs to stabilize compositions or phases that are not in equilibrium in the bulk. Reducing the crystal size to the nanoscale extends the range of compositions and crystal structures that can be achieved. New phases^64−72^ and off-stoichiometric compositions^59,73−76^ have been found to exist exclusively at the nanoscale, with no corresponding bulk counterparts. Such ICs can be explored as precursors to form stable dense solids that cannot be produced with traditional methods.

##### 2.1.1.3 Heterostructured ICs

Finally, the design of heterostructured ICs comprised of entirely distinct phases provides unique opportunities to tune the NP-based precursors; the interface between two phases can influence atomic diffusion and regulate grain growth,^77−79^ while a partial solid-state reaction can yield additional phases beyond those of the initial heterostructure.^80−82^ Finally, heterostructures provide a means to guide the nanoscale distribution of the constituents in the resulting multicomponent solids.^44,80,81^

#### 2.1.2 Surface Engineering

**Figure 3:**
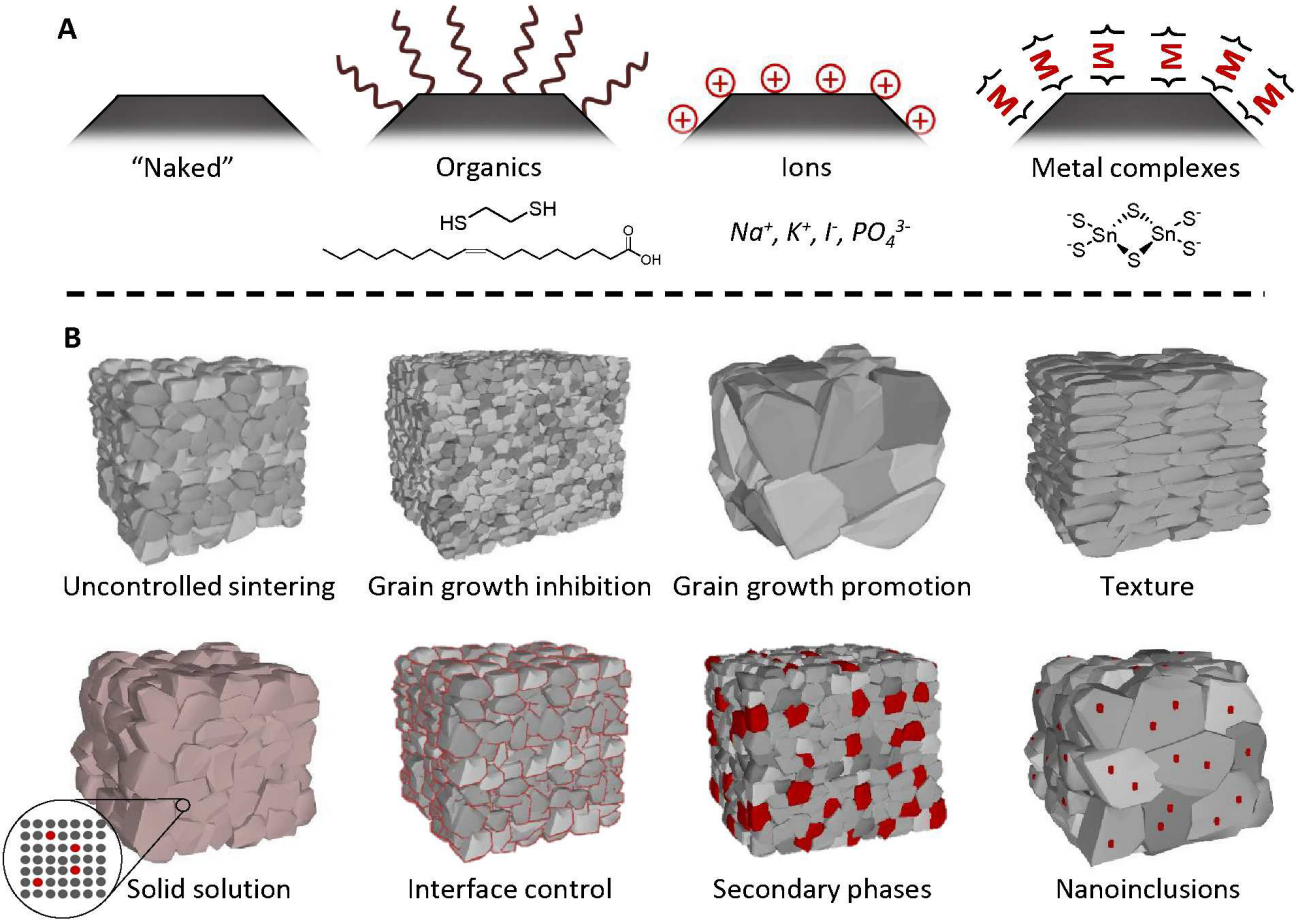
Possibilities for controlling the solid microstructure using different surface chemistries. (A) Different types of surface species that can be present. (B) Examples of different features that can be achieved in the consolidated material through surface chemistry.

Another critical feature in powder design is the NP surface. Surface atoms have a different atomic environment that can significantly alter the particle’s surface energy and reactivity,^83^ both crucial parameters for the transformation that occurs during consolidation. The surface chemistry of the NPs is comprised of two different structural features: first, the termination atoms of the NPs, which are usually determined by the synthesis, and second, the adsorbates connected to the under-coordinated termination atoms.^84,85^ The adsorption of species on the surface of the NPs occurs during synthesis or during postsynthetic treatments; such adsorbates can vary from molecules with long aliphatic chains to molecular or ionic species.^29,86^ Such surface species can be viewed as an added tunable feature in guiding sintering and solid-state reactions during consolidation rather than an inescapable detriment (Figure 3).

##### 2.1.2.1 Organic Surface Species

Most common colloidal synthetic routes yield NPs with long hydrocarbon chains, such as fatty acids, amines, or thiols.^29^ If used directly, such hybrid organic–inorganic NPs convert into composites with a carbon-containing phase.^87,88^ Besides, such ligands and their decomposition products can provide barriers, hindering atomic diffusion and therefore affecting grain growth and solid-state reactions during consolidation. Calcination in a reducing or inert atmosphere is a common procedure to remove these ligands.^89,90^ However, the calcination of long hydrocarbon chains yields graphitic carbon that can pin the grain boundaries and inhibit grain growth.^65^ Alternatively, these ligands can be exchanged for smaller, more volatile molecules such as ethylenediamine,^91^ ethylendithiol,^92^ or pyridine,^93^ which are easier to remove during calcination. Such volatile ligands, conversely, can promote grain growth.^94^ Finally, phosphine and thiols^47,48^ can be used to introduce atomic impurities (P or S) in the final solid, simultaneously doping it and potentially affecting its crystallinity.^22^

##### 2.1.2.2 Inorganic Surface Species

An alternative to organic ligands that bypasses the problem caused by residual carbon is the use of inorganic ligands. Inorganic molecules and ions were previously used to exchange the native organic ligands and functionalize IC surfaces to produce dense materials where the ligand could (i) be converted into an inorganic matrix encapsulating the ICs,^95^ (ii) react with the IC to yield a new phase,^96^ (iii) promote crystal growth,^97−99^ and (iv) introduce secondary phases^98^ or atomic impurities.^100^ Chalcogenidometalates (Sn_2_S_6_^4–^, In_2_Se_4_^2–^, and CdTe_22_^–^),^101,102^ halometallates (PbCl_3_^–^ and InCl_4_^–^),^89,103,104^ oxoanions (PO_4_^3–^ and MoO_4_^2–^),^105^ chalcogenides (S^2–^, Se^2–^, and Te^2–^),^43,44^ and halide (Cl^–^ and I^–^)^89,106^ and pseudohalide (CN^–^, SCN^–^, and N_3_^–^)^104,107^ ions are some examples of the continuously expanding collection of inorganic molecules used for surface functionalization. Many of these ligands have been successfully employed to improve the conductivity in nonsintered NP-based solids,^104,108−111^ but their effect on consolidation remains mostly unexplored.

##### 2.1.2.3 “Naked” Surfaces

The idea behind surface functionalization is to replace the native ligands with other adsorbates that have a specific function.^112^ A different direction to explore with solution-processed NPs is to create “naked” surfaces similar to powders produced by solid-state techniques like ball milling. It should be noted that truly “naked” surfaces actually do not exist in solution. In order to be colloidally stable, particles need either to be sterically stabilized by bulky ligands or electrostatically charged, inevitably leading to the coprecipitation of ions when the particles are removed from solution.^86^ However, if suitable species are placed on the surface in combination with mild thermal treatments, “naked” surfaces might become possible. For example, native organic ligands can be displaced or exchanged by small ionic adsorbates,^113^ such as hydroxide or hydrides.^114^ Lewis base ligands can also detach by forming adducts with Lewis acids such as BF_3_,^115^ and Meerwein’s salts are known to introduce a small alkyl group to the coordinating atom on the ligand, detaching it from the surface.^116,117^ Although “naked” surfaces might not seem interesting to explore, they can be used to overcome the problems caused by native ligands. Moreover, the possibility of creating naked surfaces is necessary to decouple the effects of surface adsorbates from other features of the particles and ultimately establish relationships between the properties of the NP-based precursor and those of the consolidated solids.

#### 2.2.3 NP Array Configuration

**Figure 4:**
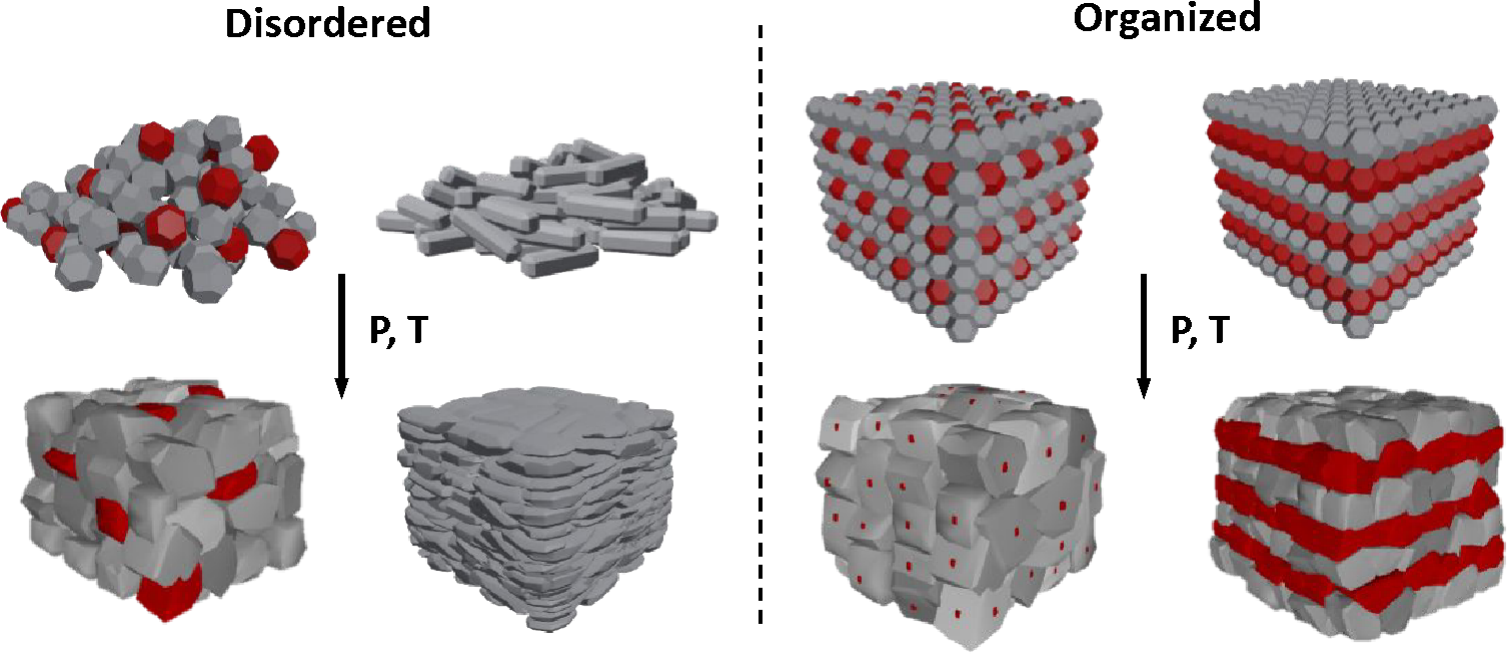
From nanoparticle arrays to consolidated solids. The distinct organization of particles prior to consolidation could lead to very different microstructural features. Random organization of the NP powder can lead to homogeneously distributed secondary phases or even solids with texture. Organized NP arrays go further and could eventually induce order in the consolidated solid.

One additional possibility that well-defined NPs offer as precursors is the ability to to control their organization, i.e., to produce NP arrays with a predetermined configuration. Managing the layout of the particles allows the initial transformation to be guided between juxtaposed particles^118,119^ and juxtaposed particle supercrystals.^120^ The production of structured powders can enable the production of complex macroscopic inorganic solids with unprecedented control of the phase and composition at multiple length scales (Figure 4).

##### 2.1.3.1 Random NP Array

The development of well-defined nanocomposites has been one of the most successful strategies of introducing scattering centers for phonons with a minimum impact on the charge carrier mobility.^121,122^ While spinodal decomposition has been successful in the production of this kind of materials, it fails to control the nanoinclusion’s size, shape, and composition. Alternatively, NPs of different types have been mixed to produce multicomponent inorganic solids.^12^ The large possibilities described above for the IC and the surface species highlight the versatility of this approach, where a practically unlimited number of solids can be made by simply blending different types of NPs. However, NPs tend to segregate into clusters of the same size, shape, or composition, leading to heterogeneity in the consolidated solid.^123^ To produce randomly yet homogeneously distributed second-phase nanoinclusions embedded in a dense matrix, it is necessary to produce a powder where different types of NPs are well mixed. Some routes to explore include blending NPs of different sizes^49,124,125^ and functionalizing the NP surfaces to negatively and positively charge each type of NP and guide their agglomeration into powder form.^126,127^

##### 2.1.3.2 Highly Ordered NP Superlattices

Finally, one of the most distinctive possibilities is using NP superlattices as precursors. High-quality particles, i.e., those with a highly homogeneous distribution of size, shape, and composition, have enabled the design of solid materials due to their assembly into long-range-ordered superstructures resembling atomic crystal lattices.^59,128,129^ Work on lead chalcogenides has even shown the possibility of epitaxially interconnecting particles to form porous single crystals by controlling ligand desorption.^130^

The chemical transformations from well-organized powders into macroscopic solids could be used to induce periodicity in nanoinclusions (Figure 4) or to create super structures similar to those developed by molecular beam epitaxy^131,132^ at a much lower cost and on a large scale. Clearly, such a level of organization in the powder is incredibly challenging to achieve, and intensive research is being carried out to develop assembly strategies that allow the large-scale 3D organization of particles. However, even in the case that only powders with a limited degree of controlled organization can be made, the resulting solids could have unique architectures with unexpected transport properties.

### 2.3 Reducing Cost: From Materials to Devices

A final advantage brought by solution processing is the opportunity to reduce the production costs both of the materials and the devices.

#### 2.2.1 Reducing the Synthetic Cost

Compared with traditional solid-state methods, solution-processed thermoelectric materials are produced in much less demanding conditions. Since diffusion in solution occurs orders of magnitudes faster, shorter times are needed compared to those in solid-state reactions. Moreover, the syntheses are usually performed at relatively low temperatures (below 350 °C), reducing the energy consumption of the process. Furthermore, as the reactions can be controlled to nucleate and grow the desired compound, solution methods usually require lower reagent purities, leaving behind possible side products, unreacted species, and the solvent, which can be separated after the synthesis.

Another important factor in reducing the cost is the solvent. Water is the most inexpensive and environmentally friendly solvent. However, it limits the synthesis temperature range and has oxidative and hydrolyzing abilities, restricting the use of very strong reductants that may be necessary. Other polar solvents, as well as the use of autoclaves, have been used to overcome these limitations. These synthetic methods are known as solvothermal and hydrothermal and have been extensively used by the thermoelectric community.^25,26,133−135^ However, precisely controlling the particles’ characteristics is much more difficult in these methods.

#### 2.2.2 Additive Manufacturing

Typically, fabricating a thermoelectric device requires dicing, bonding, and assembling multiple semiconductor *legs*. Solution processing has the potential to reduce the cost of thermoelectric devices by employing cheaper fabrication techniques. Additive manufacturing (3D printing) techniques can decrease the production cost and allow movement away from the planar geometry of conventional thermoelectric devices.^112,136−138^ However, to produce 3D-printed thermoelectric devices, it is necessary to develop thermoelectric materials in the form of an ink with very specific rheological properties that enable a proper flow for particle deposition yet maintain the structural integrity of the printed pattern.^139^ Rheological properties are tightly related to particle characteristics such as size, size distribution, and surface chemistry.^139,140^ Therefore, using solution-processed NPs with well-curated properties can be the key to establishing this technology for thermoelectrics; as we described above, there are plenty of possibilities for controlling particle properties and their surface chemistry.

### 2.4 Controlled Porosity in Inorganic Solids

So far, we have emphasized that high-density bulk materials are sought because porous materials do not provide high performances and the transport is difficult to interpret. One of the problems in understanding transport properties in porous materials is that the pores are generally uncontrolled in distribution, size, and shape. This can be changed by employing well-curated NP-based precursors, where porous materials with well-defined pore densities and structures can be produced. One possibility is to synthesize hollow NPs as precursors and fuse them into 3D grid-like structures with uniform pores by applying mild consolidation conditions that do not destroy or modify the voids. Similar porous structures could also be prepared using epitaxially fused NP superlattices where the particles are connected only in certain facets, leaving empty spaces between the different connected NPs. Hollow NPs can also be employed to produce porous materials with different densities by encapsulating them into matrices. This can be achieved by functionalizing the hollow structures with a precursor that would yield the matrix material during the thermal treatments or by blending the hollow NPs at different ratios with other types of NPs that represent the matrix material.

Overall, there is much room to explore in the field of porous semiconductors. Beyond the search for new exciting transport phenomena, porous materials are attractive because they help to reduce module costs and weight, since less material is used.

## 3 The Challenges

This perspective presents the idea that by carefully engineering NP-based precursors, tailored dense solids with targeted features can be prepared. Yet, this approach is still in the very early stages of development and is therefore very challenging. Foremost, it is necessary to establish correlations between NP properties and structural properties of the consolidated material. Such correlations will turn the *by chance* synthesis of dense inorganic materials into a *by design* synthesis, where the desired features can be obtained from carefully designed NPs. This task is extremely arduous. One of the major limitations to establishing such causality is the lack of detailed structural information on both the NP-based precursors and the dense solid. Furthermore, in most cases, we ignore the chemical transformations that occur during the consolidation, which can eventually lead to an incorrect interpretation of the physical and chemical changes that the material undergoes.

While trying to exploit all the possibilities solution-processed thermoelectric materials can bring to the table, certain peculiarities of the process need to be taken into account. Solution-processed materials tend to be more unstable than those prepared by solid-state methods. This needs to be remedied before acquiring the data so reproducible and accurate measurements are reported. Additionally, most solution-processed materials show temperature-activated charge transport, indicating the presence of energy barriers that render them less efficient than materials prepared by conventional solid-state routes.

### 3.1 In-Depth Characterization of NP-Based Precursors and the Consolidated Solid

#### 3.1.1 Characterization of the NP-based Precursors

The comprehensive characterization of the NP-based precursor requires the size, shape, crystal structure, composition, surface termination, and surface adsorbates of the NP and the NP array organization in the powder to be determined. The standard characterization of NPs includes electron microscopy, which directly examines the NP size and morphology;^141^ UV–vis spectroscopy, which in some cases can provide information on NP size and size dispersion;^57,142,143^ and X-ray diffraction (XRD), which is used to determine the crystallographic phase and to estimate the particle size (Scherrer analysis).^144^ Furthermore, the sizing and aggregation of NPs in solution can be analyzed with dynamic light scattering (DLS).^145^ However, these characterization methods are sometimes insufficient to correctly describe the structure of the NP; therefore, further techniques must be employed. Among the techniques, the pair distribution-function analysis of high-quality XRD data can provide an atomically precise description of the NPs, including size, and the vacancy occupation, even for NP with low crystallinity.^146^ Electron diffraction techniques can be used to investigate the crystallographic phase with a high spatial resolution,^147,148^ and aberration-corrected electron microscopy can allow the mapping of atomic terminations.^141,149,150^ Small-angle X-ray scattering (SAXS) can deal directly with the precursor in solution and in the powder form and is sensitive to the NP size and assembly.^151^

The accurate determination of the composition of solution-processed materials is crucial because the real composition often varies from the nominal one, as opposed to traditional solid-state methods. It has been extensively shown that minor changes in stoichiometry can have a massive impact on the properties of the final solid.^152,153^ The composition of NPs is often determined by energy-dispersive X-ray spectroscopy (EDX), but this technique lacks the accuracy required for a proper rationalization of the process. Optical emission spectroscopy (ICP-OES) and mass spectroscopy have the necessary elemental sensitivity but do not provide information on the distribution of measured elements. Therefore, techniques that are sensitive to the atomic environment and the surface should be employed, such as X-ray absorption spectroscopy,^154^ and X-ray photoelectron spectroscopy (XPS), which are also sensitive to the oxidation state of the elements^155^ and the presence of oxide.^156,157^

Identifying the surface species is crucial to understanding their role during the formation of the solid.^83^ First, the assessment of the surface chemistry is often done with Fourier-transform infrared spectroscopy, which can easily indicate if organic ligands are present.^158^ However, this technique cannot provide all the necessary information needed to deduce the surface structure and does not work for most bound small molecules. Nuclear magnetic resonance (NMR) can be used to identify and quantify both bound organic ligands and solvated species.^47,48,159−161^ Solid-state NMR, on the other hand, can provide information on the atomic structures of NP surfaces.^162^ DLS combined with ζ-potential measurements allows the charge of colloids to be determined, which helps to elucidate the structure of the electrical double layer in charged NPs.^159^ Grazing incidence X-ray absorption and photoelectron spectroscopies can also be used to disclose how NPs are on the surface.^163^

Despite the plethora of methods available to study the surface chemistry of NPs, it has been proven that some surface species remain undetectable. For example, observing ionic groups intercalating between organic molecules is complicated.^163,164^ If the surface is not analyzed with the right tools, there is a considerable risk of overlooking certain species, leading to an incorrect interpretation of the observed phenomena. This represents a challenge, and careful considerations need to be made to trace all possible elements that may end up on the particle surface by considering both the precursors used for the NP synthesis and the surface treatments used.^165^

#### 3.1.2 Characterization of the Consolidated Solid

The basic structural characterization of consolidated thermoelectric materials is usually performed by XRD using laboratory sources. XRD allows phase identification, provides information on crystallographic texture^166^ and strain, and in some cases allows an estimation of the doping level using Vegard’s law.^167^ While such information is of high value, XRD does not provide comprehensive information on the different defects present in the material, and various techniques must be employed. The characterization of choice depends on the characteristic length scale and the dimensionality of the defects; thus, it is important to include other techniques in the standard characterization of samples that might disclose the presence of sometimes unexpected defects.

##### 3.1.2.1 0D

Point defects can be evaluated using neutron diffraction,^168^ synchrotron XRD,^169^ or Cs-corrected high-resolution transmission electron microscopy (TEM) and atomic resolution electron energy loss spectroscopy.^170−173^ Another technique that is capable of analyzing point defects is atom probe tomography (APT), which reconstructs compositional maps of small volumes with an atomic spatial resolution, below parts per million chemical sensitivity, and equal sensitivity to all elements.^174^ APT can reveal the spatial distribution of dopants, including light elements such as sodium,^175^ which are difficult to detect by other means and give insights regarding the dopant efficiency.^18^ Further techniques, still relatively unexplored, are positron annihilation spectroscopy (PAS) and deep-level transient spectroscopy (DLTS).^176^ PAS is predominantly used to quantify vacancies, but is not restricted to it and can shed light on the type and relative concentration of defects.^177−179^ DLTS can clarify the relationship between the charge carrier concentration and point defects.^176^

##### 3.1.2.2 1D

Line defects are typically analyzed by advanced TEM or APT. From TEM, dislocation densities^180^ can be estimated directly, whereas in APT dislocations are only indirectly identified if they have a different composition.^181,182^

##### 3.1.2.3 2D

Planar defects (grain boundaries, phase boundaries, twin boundaries, and stacking faults) can be investigated by TEM, APT, Kelvin probe force microscopy (KPFM), and electron backscatter diffraction (EBSD). Like dislocations, grain boundaries can be directly imaged in TEM.^183^ APT data can indicate if there is a compositional discontinuity at the grain boundaries, and it is a very powerful technique for identifying grain boundary complexions,^184^ which can be elusive with TEM. KPFM can also indirectly provide information about grain boundaries by detecting variations in the interfacial barriers due to charge accumulation at grain boundaries. EBSD is usually conducted in a scanning electron microscope (SEM) and gives valuable information on the type of grain boundaries (high angle or low angle), their respective concentrations, texture, and strain.

##### 3.1.2.4 3D

Bulk defects encompass nanoinclusions and pores. PAS can be used to detect pores and distinguish between different sizes. Nanoinclusions can be analyzed by XRD, TEM, SEM, and APT. While APT can give the size, shape, distribution, and chemical nature of such precipitates, TEM can also provide information about the crystal structure and the strain between the precipitate and the matrix. Moreover, gas physisorption isotherms are among the most common experiments used to study porous samples, including pore volume, pore size distribution, and surface area, although their use in thermoelectric materials is very limited.^185,186^

#### 3.1.3 Characterization of the Transformation from NP-Based Precursors to Dense Solids

In some cases, a complete characterization of the NP-based precursor and the consolidated solid is not enough to understand how the transformations occur and establish causality. In those cases, directly examining the transformation of NP-based precursors into the consolidated solid can unveil the presence of intermediates and metastable phases hidden in the process and ultimately explain the transformation. Among the techniques that are useful for studying the consolidation process, we can mention temperature-dependent TEM, which uses a heating stage to follow neck formation between particles,^187^ changes in particle shape, size, and composition or the crystallographic phase,^188^ and the structural changes and structural arrangements of pores;^189,190^ XRD coupled with a reaction chamber, which is used to evaluate the evolution of the crystal structure,^189^ disorder, grain size, crystallographic texture, phase transitions,^191,192^ and side reactions;^49^ SAXS temperature-dependent measurements in a controlled atmosphere, which is used to follow grain size and shape, porosity (pore structure and distribution), and the presence of thick boundaries;^193−195^ differential scanning calorimetry, which is used to track exothermic or endothermic events to identify reaction points or phase transitions;^196^ mass spectroscopy and gas chromatography, which are used to analyze the gas that evolves during the powder transformation;^197^ and thermogravimetry, which is used to evaluate mass changes during heating that might include the formation of volatile reaction products or reactions with the gas phase.^191^

However, the use of *in situ* methods to characterize the transformation of the NP-based precursor into the consolidated solid is limited. For proper characterization, the consolidation conditions must be replicated in the measurement (e.g., high temperature, pressure, and inert atmosphere), and the time scale of the measurements has to match the time scale of the processes being investigated. A way around this latter problem is to quench the processes at intermediate stages and analyze them separately.

The techniques listed here do not cover all the existing characterization tools but give an idea of the most useful for studying the NP-based precursor, the consolidated solid, and the transformation of one into the other. Using such a comprehensive set of techniques requires a large set of expertise for data acquisition and analysis. Moreover, some techniques are not readily available in standard laboratories, either because of cost or infrastructure limitations. Therefore, the only way to move forward in expanding the collection of characterization techniques is through collaboration with specialized scientists.

### 3.2 Problems Associated with the Solution Synthesis of the Powder

**Figure 5:**
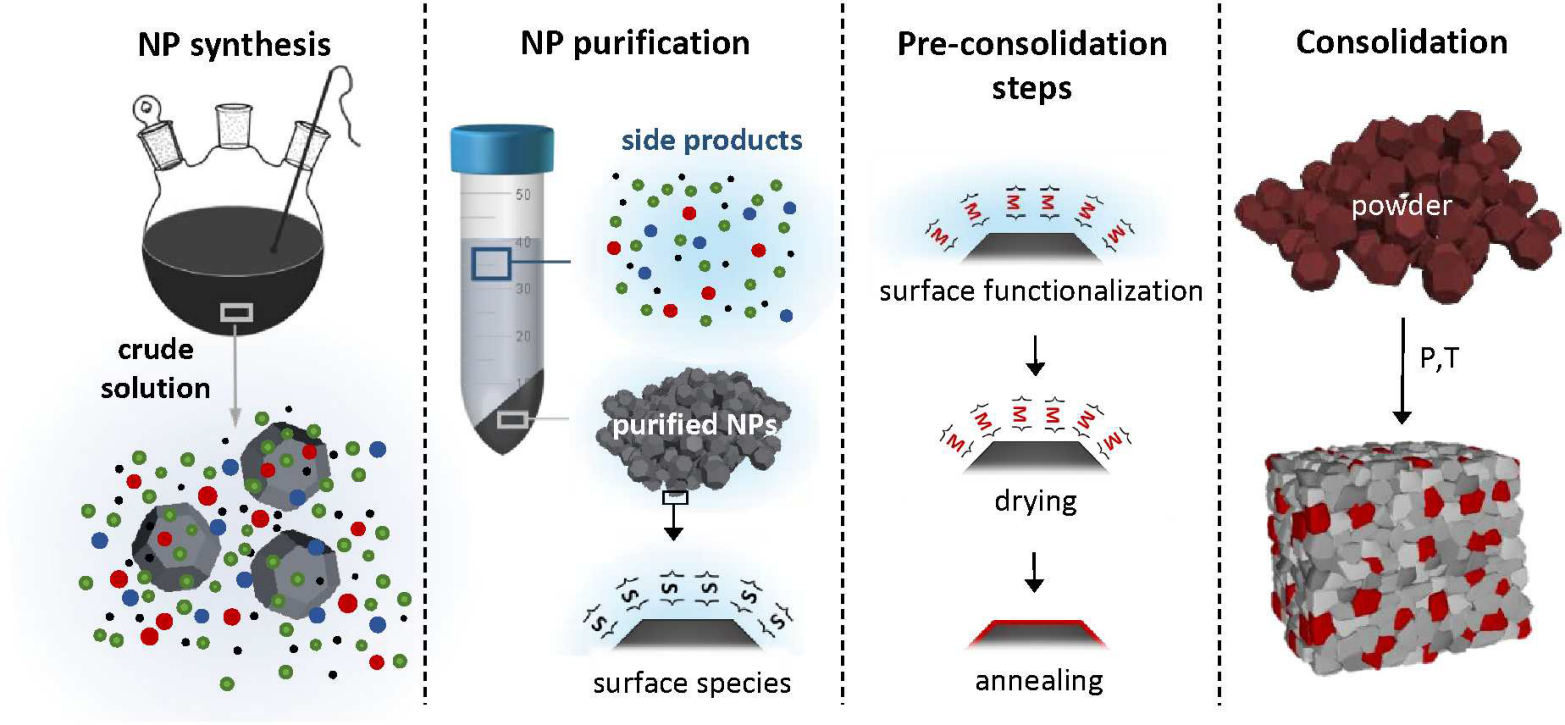
Schematic of the multistep process for preparing dense semiconductors from NPs synthesized in solution. Different from the preparation of the powder by mechanical methods, solution processing involves many steps, for example, the separation of the solvent and byproducts and surface functionalization. These “extra” steps allow for better control of the NP-based precursor but also carry new challenges.

The multistep process of preparing dense semiconductor solids from solution-processed NPs (Figure 5) not only offers opportunities to tune the NPs but it also has associated challenges, such as low reproducibility, instability of the materials, low densities, and oxidation.

#### 3.2.1 Reproducibility

Low reproducibility of solution-processed materials is a persistent issue, especially among different laboratories. The first reason is the lack of the careful handling of the NP-based precursor. For example, small differences in the chemicals used^198−200^ or the purification conditions (duration, particle concentration, solvent, cycles, and environment) can yield different types and amount of impurities that impact the material microstructure.^201^ The same can happen with the surface treatments and drying steps; the presence of surface adsorbates and the effect of volatile species influence the formation of the solid and even its stability.^86^ Another source of discrepancy among samples is the homogeneity of the powder prior to annealing. To warrant reproducibility, it is mandatory to be very methodical, to develop a deep understanding of the different steps by detailed characterization, and to report all the details of every processing step.

#### 3.2.2 Density Control

To date, the role of pores in thermoelectric materials remains unclear. Effective medium theories predict that the increase in *zT* caused by the reduction of the thermal conductivity is canceled by the reduction of the electrical conductivity, hence it does not have any effect on the thermoelectric figure of merit.^202−204^ This should hold true if the pores lead to inert grain boundaries.^205,206^ However, if that were the case, more work would be done on producing high-performance materials with large porosity to deliver lighter and cheaper devices.^207^ Yet, the lack of work on materials with reproducible porosity and the absence of theories that explain transport in porous media make acquiring reliable data and evaluating it difficult, particularly for thermal conductivity. Therefore, the thermoelectric community seeks to fabricate solids with densities as close to their theoretical values as possible. When the powder is produced in solution, densities are often lower than those achieved in solid-state methods, up to around 80% of the theoretical density.^65^ This can be attributed to the remaining solvent or adsorbed species that decompose and evaporate during the sintering step, creating pores. To avoid this, the most common approach is to anneal the powder at temperatures above the evaporation or decomposition temperature of the bound molecules and solvent (≥350 °C), although this is not always done.

#### 3.2.3 Oxidation

The use of solvents for the synthesis and purification of the NPs results almost unavoidably in the presence of oxygen and, consequently, in the (at least partial) oxidation of the NPs.^208^ Different solvents have distinct oxidative properties. For instance, the concentration of dissolved oxygen in nonpolar solvents can be orders of magnitude higher than in water or polar organic solvents.^209,210^ This does not necessarily translate into more oxidation; the self-ionization of protic solvents, redox stability zones, and solvent reorganization energies can affect the oxidation potential and rate.^211^ In water, dissolved atmospheric CO_2_ slightly increases the acidity, further promoting oxidation. An interesting example that reflects the complex role of solvents is the case of Cu nanowires, where polar solvents were effective in minimizing oxidation but nonpolar solvents led to heavily oxidized samples.^212^

Dissolved oxygen is not the only source of NP oxidation, but it is definitely the main one due to its ubiquity (21% in air), leading to performance degradation. Moreover, due to the large surface-to-volume ratio, surface oxidation remains challenging despite efforts to mitigate this effect.^213^ The high reactivity allows NPs to easily react with oxygen to form corresponding oxides. Even a small layer of oxygen on the surface of the NPs results in a high oxide volume ratio. NPs of several materials that do not oxidize in atmospheric conditions in the bulk have been reported to undergo oxidation, such as noble metal NPs (Au and Ag)^214,215^ and group II–IV NPs.^216^

This inherently leads to the incorporation of oxides into the consolidated material, which affects the thermoelectric performance.^217^ In the case of CdSe, reports revealed that surface oxidation occurs via two mechanisms: physisorption, which can be reversible, and chemisorption, which results in the NP being etched into smaller sizes.^218−221^ To date, there are several strategies for minimizing oxidation throughout solution processing. Such methods include purging solvents with inert gas, conducting the process under inert atmospheres, using dry air-free solvents, and annealing under a reducing environment.^222^

#### 3.2.4 Instability

Another problem of solution-processed thermoelectric materials is their instability, which is visible by different trends during the heating and cooling cycles of the temperature-dependent transport measurements.^223^ It can take multiple heating and cooling cycles for some solution-processed materials to report the same transport values, indicating that the materials still undergo transformations in the initial cycles. Such behavior can be related to the presence of volatile species trapped in the material that slowly escape during the measurements, especially at high temperatures. Introducing additional post-consolidation thermal treatments can solve this issue.^98^

### 3.3 The Presence of Energy Barriers

When comparing the transport behaviors of solution-processed materials with those of their solid-state counterparts, solution-processed TE materials generally show lower electrical conductivities at room temperature. Moreover, the temperature-dependent trend is that the conductivity increases with temperature instead of decreasing. Some examples of NP-based solids that behave differently from the bulk ones include PbS,^49,224^ PbTe,^144,225,226^ and Ag_2_Te-PbTe.^123,227^ Such thermally activated electrical conductivity represents a challenge, as it seriously impairs the *zT* of the solution-processed thermoelectric material at low temperatures. While the performance may be higher at high temperatures, the low performance at low temperatures deteriorates the average *zT*, which determines the device efficiency. Hence, thermally activated electrical transport represents a severe challenge for low-temperature generators and cooling applications.

In intrinsic semiconductors, thermal activation of charge carriers leads to electrical conductivities that increase with temperature. However, inorganic thermoelectric materials^228,229^ are heavily doped, so the thermal activation comes from the energy barriers related to the defects. In particular, there is a high density of interfacial defects present in NP-based materials, such as pores, the presence of oxide, graphitic carbon from decomposed surface adsorbates, and grain boundary interfacial phases (also known as grain boundary complexions).^230−232^ To overcome this challenge, it is necessary to study how specific defects affect the electrical conductivity and develop strategies to design defects and, in particular, interfacial phases that are beneficial for the charge transport yet scatter phonons effectively.

## 4 Good Practices

As mentioned previously, one of the major issues is reproducibility among different laboratories. One of the reasons for this problem is the lack of experimental details in the publications. While it is common to report the whole process in general lines, details considered irrelevant are left behind without exploring how important those parameters actually are. In the case of solution-processed thermoelectric materials, this is even more significant, as multiple steps are involved (Figure 5). Therefore, thoroughly detailed reports are crucial to driving reproducibility among research groups. This will ultimately facilitate and speed up thermoelectric research.

In the following lines, we describe a guideline for best practices to promote reproducibility among different laboratories:

1. **Reactants used**. The first problem can be found in the reporting of the chemicals used. It is critical to not only list all the chemicals employed but also to specify if reactants are synthesized from commercial chemicals (indicating their purity and brand) or bought and whether they are further purified.
2. **Synthetic conditions**. Most reports described the synthetic conditions by reporting only some parameters such as atmosphere, temperature, and time steps. Missing details include the injection rate of precursors, information about temperature control (PID parameters, heating ramps and profiles, cooling rate, heating source, etc.), data on scalability, stirring characteristics (type and speed), and an image of the experimental setup.
3. **Particle purification process**. When the powder is produced in solution, it usually requires a series of purification steps, where the particles are separated from the solvent, unreacted species, and soluble byproducts (Figure 5). Most works only report the purification as “the particles have been rinsed multiple times with X and Y solvents”. However, no volumes, number of cycles, or rinsing conditions are specified. The best practice is to report all these experimental details, as well as explain why the solvents are chosen and how the NPs are dried. Small impurities left behind due to inadequate purifying processes can significantly influence the structure and performance of the final material.
4. **Surface treatment methodology**. Concerning surface functionalization, it is important to specify not only the solvent, the concentration of the NPs, and the reactants used but also the volume. Depending on whether these reactions are highly endo or exothermic, direct up- or down-scaling, respectively, might not work. Furthermore, stirring conditions and further purification should be mentioned. As important as the treatment itself, the handling of the NPs after functionalization is critical (washing, storage life, storage solvent, atmosphere, temperature, excess surfactants used for colloid stability, pH, etc).
5. **Preconsolidation and postconsolidation treatments.** Mild annealing treatments are usually performed to remove adsorbed solvent or to thermally decompose the surface adsorbates. In most reports, there is no indication of the specific temperature profile used, and in the best cases only the maximum temperature, atmosphere, and time are reported. However, heating and cooling rates and the gas flow rate impact the structure and composition of the final material and hence must be reported. Additionally, the experimental setup of the furnace (i.e., in or outside a glovebox) and sample handling during loading and unloading from the oven should be detailed.
6. **Study of potential surface species**, even for those cases where it is assumed the NPs are “naked”. So-called surfactant-free methods render particles without organic molecules on the particle surface, and the particle’s surface is usually considered “naked”. Thus, only a drying step is performed before consolidation. Despite the fact that the surface is considered “clean” or “naked”, different species might be adsorbed on the particle surface depending on the particle composition and surface termination^94,233,234^ (see section 3.1.1).
7. **Stability testing.** To ensure the stability of the material, measuring multiple heating and cooling cycles are required until the cycles coincide. Moreover, thermogravimetric measurements to monitor changes in the sample mass as a function of temperature and control measurements, e.g. XRD, after each temperature-dependent measurement can complement this. Stability tests must always be available, at least as supporting information, and it is the responsibility of authors to publish them and of reviewers and editors to demand them.

## 5 Outlook

**Figure 6:**
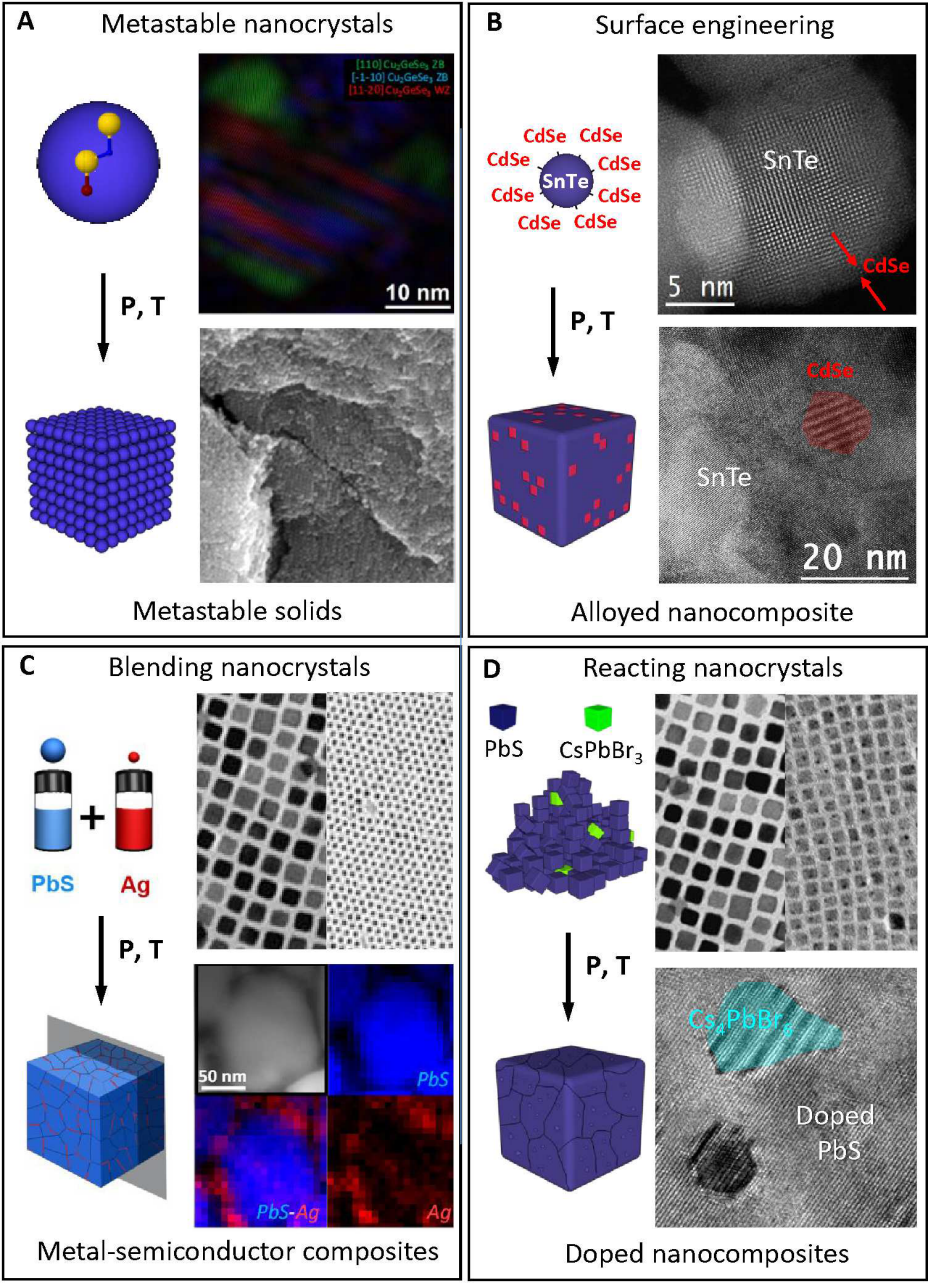
Examples of polycrystalline solids obtained by the reaction of precisely design NPs. (A) NPs with metastable phases yield macroscopic metastable solids. Reproduced from ref (65). Copyright 2012, American Chemical Society. (B) Surface-modified NPs generate nanocomposites with a tuned stoichiometry. Reproduced with permission from ref (102). Copyright 2019, American Chemical Society. (C) Blending metallic and semiconductor NPs creates doped nanocomposites. Reproduced from ref (12). Copyright 2016, Nature Publishing Group. (D) NP reactions produce doped nanocomposites. Reproduced from ref (49). Copyright 2021, American Chemical Society.

The use of well-defined NPs as precursors for the production of dense thermoelectric materials has been barely explored, yet very interesting materials with unique properties have already been reported (Figure 6).^12,49,65,102^

This perspective describes an idea that combines the best of both worlds. We propose a synthetic strategy that aims to translate the control at the NP level to dense solids with optimized properties and functionalities. Maximizing the control over the NP properties would allow the chemical transformation to be guided toward specific features in the consolidated solid, with the ultimate goal of achieving the highly sought s*ynthesis by design.*

The constant progress in NP synthesis, surface chemistry, and assembly, together with an understanding of diffusion and solid-state reactions at the nanometer scale, provides the grounds to pursue this synthetic approach for thermoelectric materials. However, it also can be extended to other fields where tailoring the microstructure is the key to breakthrough performances, including photovoltaics, catalysis, etc.

S*ynthesis by design* is slowly becoming a reality in organic chemistry, realizing the ultimate goal of synthesizing almost any target small molecule. Key to this development were, among others, retrosynthetic analysis and a deep mechanistic understanding of chemical pathways that transform reactants into products. For inorganic solids, however, *synthesis by design* remains a fairy tale.

We are aware that, at the moment, the proposed synthetic strategy is just a concept with many unknowns. However, we must implement creative strategies to explore and mass-produce novel crystalline inorganic solids with new, optimized, and even exotic properties.

## References

[ref1] Beretta D.; Neophytou N.; Hodges J. M.; Kanatzidis M. G.; Narducci D.; Martin-Gonzalez M.; Beekman M.; Balke B.; Cerretti G.; Tremel W.; et al. Thermoelectrics: From History, a Window to the Future. Mater. Sci. Eng. R Reports 2019, 138, 100501 10.1016/j.mser.2018.09.001.

[ref2] Energy Flow Charts. Lawrence Livermore National Laboratory. https://flowcharts.llnl.gov/commodities/energy (accessed 2022-06-27).

[ref3] Haras M.; Skotnicki T. Thermoelectricity for IoT – A Review. Nano Energy 2018, 54, 461–476. 10.1016/j.nanoen.2018.10.013.

[ref4] Disalvo F. J. Thermoelectric Cooling and Power Generation. Science 1999, 285, 703–706. 10.1126/science.285.5428.703. 10426986

[ref5] Snyder G. J.; Soto M.; Alley R.; Koester D.; Conner B. Hot Spot Cooling Using Embedded Thermoelectric Coolers. In Proceedings of the Twenty-Second Annual IEEE Semiconductor Thermal Measurement And Management Symposium, Dallas, TX, March 14–16, 2006; Institute of Electrical and Electronics Engineers: Piscataway, NJ, 2006; pp 135–143.

[ref6] Snyder G. J.; LeBlanc S.; Crane D.; Pangborn H.; Forest C. E.; Rattner A.; Borgsmiller L.; Priya S. Distributed and Localized Cooling with Thermoelectrics. Joule 2021, 5, 748–751. 10.1016/j.joule.2021.02.011.

[ref7] Rowe D. M. Applications of Nuclear-Powered Thermoelectric Generators in Space. Appl. Energy 1991, 40, 241–271. 10.1016/0306-2619(91)90020-X.

[ref8] Ioffe A. F.; Stil’bans L. S.; Iordanishvili E. K.; Stavitskaya T. S.; Gelbtuch A.; Vineyard G. Semiconductor Thermoelements and Thermoelectric Cooling. Phys. Today 1959, 12, 42 10.1063/1.3060810.

[ref9] Pei Y.; Wang H.; Snyder G. J. Band Engineering of Thermoelectric Materials. Adv. Mater. 2012, 24, 6125–6135. 10.1002/adma.201202919. 23074043

[ref10] Heremans J. P.; Jovovic V.; Toberer E. S.; Saramat A.; Kurosaki K.; Charoenphakdee A.; Yamanaka S.; Snyder G. J. Enhancement of Thermoelectric Efficiency in PbTe by Distortion of the Electronic Density of States. Science 2008, 321, 554–557. 10.1126/science.1159725. 18653890

[ref11] Pei Y.; Shi X.; Lalonde A.; Wang H.; Chen L.; Snyder G. J. Convergence of Electronic Bands for High Performance Bulk Thermoelectrics. Nature 2011, 473, 66–69. 10.1038/nature09996. 21544143

[ref12] Ibáñez M.; Luo Z.; Genç A.; Piveteau L.; Ortega S.; Cadavid D.; Dobrozhan O.; Liu Y.; Nachtegaal M.; Zebarjadi M.; et al. High-Performance Thermoelectric Nanocomposites from Nanocrystal Building Blocks. Nat. Commun. 2016, 7, 10766 10.1038/ncomms10766. 26948987 PMC4786643

[ref13] Luo Z. Z.; Cai S.; Hao S.; Bailey T. P.; Xie H.; Slade T. J.; Liu Y.; Luo Y.; Chen Z.; Xu J.; et al. Valence Disproportionation of GeS in the PbS Matrix Forms Pb_5_Ge_5_S_12_ Inclusions with Conduction Band Alignment Leading to High n-Type Thermoelectric Performance. J. Am. Chem. Soc. 2022, 144, 7402–7413. 10.1021/jacs.2c01706. 35420804

[ref14] Hanus R.; Agne M. T.; Rettie A. J. E.; Chen Z.; Tan G.; Chung D. Y.; Kanatzidis M. G.; Pei Y.; Voorhees P. W.; Snyder G. J.; et al. Lattice Softening Significantly Reduces Thermal Conductivity and Leads to High Thermoelectric Efficiency. Adv. Mater. 2019, 31, 1900108 10.1002/adma.201900108. 30968467

[ref15] Slade T. J.; Anand S.; Wood M.; Male J. P.; Imasato K.; Cheikh D.; Al Malki M. M.; Agne M. T.; Griffith K. J.; Bux S. K.; et al. Charge-Carrier-Mediated Lattice Softening Contributes to High ZT in Thermoelectric Semiconductors. Joule 2021, 5, 1168–1182. 10.1016/j.joule.2021.03.009.

[ref16] Roychowdhury S.; Ghosh T.; Arora R.; Samanta M.; Xie L.; Singh N. K.; Soni A.; He J.; Waghmare U. V.; Biswas K. Enhanced Atomic Ordering Leads to High Thermoelectric Performance in AgSbTe_2_. Science 2021, 371, 722–727. 10.1126/science.abb3517. 33574210

[ref17] Puneet P.; Podila R.; Karakaya M.; Zhu S.; He J.; Tritt T. M.; Dresselhaus M. S.; Rao A. M. Preferential Scattering by Interfacial Charged Defects for Enhanced Thermoelectric Performance in Few-Layered n-Type Bi_2_Te_3_. Sci. Rep. 2013, 3, 3212 10.1038/srep03212. 24225424 PMC3827612

[ref18] Yu Y.; Zhou C.; Zhang S.; Zhu M.; Wuttig M.; Scheu C.; Raabe D.; Snyder G. J.; Gault B.; Cojocaru-Mirédin O. Revealing Nano-Chemistry at Lattice Defects in Thermoelectric Materials Using Atom Probe Tomography. Mater. Today 2020, 32, 260–274. 10.1016/j.mattod.2019.11.010.

[ref19] Yuan M.; Liu M.; Sargent E. H. Colloidal Quantum Dot Solids for Solution-Processed Solar Cells. Nat. Energy 2016, 1, 16016 10.1038/nenergy.2016.16.

[ref20] García De Arquer F. P.; Armin A.; Meredith P.; Sargent E. H. Solution-Processed Semiconductors for next-Generation Photodetectors. Nat. Rev. Mater. 2017, 2, 16100 10.1038/natrevmats.2016.100.

[ref21] Zevalkink A.; Smiadak D. M.; Blackburn J. L.; Ferguson A. J.; Chabinyc M. L.; Delaire O.; Wang J.; Kovnir K.; Martin J.; Schelhas L. T.; et al. A Practical Field Guide to Thermoelectrics: Fundamentals, Synthesis, and Characterization. Appl. Phys. Rev. 2018, 5, 021303 10.1063/1.5021094.

[ref22] Kang S.-J. L. Sintering: Densification, Grain Growth, and Microstructure; Elsevier: Burlington, MA, 2005.

[ref23] Bourgès C.; Bouyrie Y.; Supka A. R.; Al Rahal Al Orabi R.; Lemoine P.; Lebedev O. I.; Ohta M.; Suekuni K.; Nassif V.; Hardy V.; et al. High-Performance Thermoelectric Bulk Colusite by Process Controlled Structural Disordering. J. Am. Chem. Soc. 2018, 140, 2186–2195. 10.1021/jacs.7b11224. 29332398

[ref24] Jin Y.; Wang D.; Hong T.; Su L.; Shi H.; Zhan S.; Wang Y.; Wang S.; Gao X.; Qiu Y.; et al. Outstanding CdSe with Multiple Functions Leads to High Performance of GeTe Thermoelectrics. Adv. Energy Mater. 2022, 12, 2103779 10.1002/aenm.202103779.

[ref25] Shi X. L.; Tao X.; Zou J.; Chen Z. G. High-Performance Thermoelectric SnSe: Aqueous Synthesis, Innovations, and Challenges. Adv. Sci. 2020, 7, 1902923 10.1002/advs.201902923. PMC7141048 32274303

[ref26] Shi X.-L.; Liu W.-D.; Li M.; Sun Q.; Xu S.-D.; Du D.; Zou J.; Chen Z.-G.; et al. A Solvothermal Synthetic Environmental Design for High-Performance SnSe-Based Thermoelectric Materials. Adv. Energy Mater. 2022, 12, 2200670 10.1002/aenm.202200670.

[ref27] Channegowda M.; Mulla R.; Nagaraj Y.; Lokesh S.; Nayak S.; Mudhulu S.; Rastogi C. K.; Dunnill C. W.; Rajan H. K.; Khosla A. Comprehensive Insights into Synthesis, Structural Features, and Thermoelectric Properties of High-Performance Inorganic Chalcogenide Nanomaterials for Conversion of Waste Heat to Electricity. ACS Appl. Energy Mater. 2022, 5, 7913–7943. 10.1021/acsaem.2c01353.

[ref28] Van Embden J.; Chesman A. S. R.; Jasieniak J. J. The Heat-up Synthesis of Colloidal Nanocrystals. Chem. Mater. 2015, 27, 2246–2285. 10.1021/cm5028964.

[ref29] Heuer-Jungemann A.; Feliu N.; Bakaimi I.; Hamaly M.; Alkilany A.; Chakraborty I.; Masood A.; Casula M. F.; Kostopoulou A.; Oh E.; et al. The Role of Ligands in the Chemical Synthesis and Applications of Inorganic Nanoparticles. Chem. Rev. 2019, 119, 4819–4880. 10.1021/acs.chemrev.8b00733. 30920815

[ref30] Lesnyak V.; Yarema M.; Miao S. Editorial: Colloidal Semiconductor Nanocrystals: Synthesis, Properties, and Applications. Front. Chem. 2019, 7, 684 10.3389/fchem.2019.00684. 31696104 PMC6817508

[ref31] Hens Z.; De Roo J. Atomically Precise Nanocrystals. J. Am. Chem. Soc. 2020, 142, 15627–15637. 10.1021/jacs.0c05082. 32804488

[ref32] Loiudice A.; Buonsanti R. Reaction Intermediates in the Synthesis of Colloidal Nanocrystals. Nat. Synth. 2022, 1, 344–351. 10.1038/s44160-022-00056-x.

[ref33] Coughlan C.; Ibáñez M.; Dobrozhan O.; Singh A.; Cabot A.; Ryan K. M. Compound Copper Chalcogenide Nanocrystals. Chem. Rev. 2017, 117, 5865–6109. 10.1021/acs.chemrev.6b00376. 28394585

[ref34] Steimle B. C.; Fenton J. L.; Schaak R. E. Rational Construction of a Scalable Heterostructured Nanorod Megalibrary. Science 2020, 367, 418–424. 10.1126/science.aaz1172. 31974249

[ref35] Liu J.; Zhang J. Nanointerface Chemistry: Lattice-Mismatch-Directed Synthesis and Application of Hybrid Nanocrystals. Chem. Rev. 2020, 120, 2123–2170. 10.1021/acs.chemrev.9b00443. 31971378

[ref36] De Roo J. Chemical Considerations for Colloidal Nanocrystal Synthesis. Chem. Mater. 2022, 34, 5766–5779. 10.1021/acs.chemmater.2c01058.

[ref37] Efros A. L.; Brus L. E. Nanocrystal Quantum Dots: From Discovery to Modern Development. ACS Nano 2021, 15, 6192–6210. 10.1021/acsnano.1c01399. 33830732

[ref38] Kumar A.; Jeon K. W.; Kumari N.; Lee I. S. Spatially Confined Formation and Transformation of Nanocrystals within Nanometer-Sized Reaction Media. Acc. Chem. Res. 2018, 51, 2867–2879. 10.1021/acs.accounts.8b00338. 30346727

[ref39] Zhong H.; Lo S. S.; Mirkovic T.; Li Y.; Ding Y.; Li Y.; Scholes G. D. Noninjection Gram-Scale Synthesis of Monodisperse Pyramidal CuInS_2_ Nanocrystals and Their Size-Dependent Properties. ACS Nano 2010, 4, 5253–5262. 10.1021/nn1015538. 20815394

[ref40] Weidman M. C.; Beck M. E.; Hoffman R. S.; Prins F.; Tisdale W. A. Monodisperse, Air-Stable PbS Nanocrystals via Precursor Stoichiometry Control. ACS Nano 2014, 8, 6363–6371. 10.1021/nn5018654. 24840645

[ref41] Akkerman Q. A.; Park S.; Radicchi E.; Nunzi F.; Mosconi E.; De Angelis F.; Brescia R.; Rastogi P.; Prato M.; Manna L. Nearly Monodisperse Insulator Cs_4_PbX_6_ (X = Cl, Br, I) Nanocrystals, Their Mixed Halide Compositions, and Their Transformation into CsPbX_3_ Nanocrystals. Nano Lett. 2017, 17, 1924–1930. 10.1021/acs.nanolett.6b05262. 28196323 PMC5345893

[ref42] Ortega S.; Ibáñez M.; Liu Y.; Zhang Y.; Kovalenko M. V.; Cadavid D.; Cabot A. Bottom-up Engineering of Thermoelectric Nanomaterials and Devices from Solution-Processed Nanoparticle Building Blocks. Chem. Soc. Rev. 2017, 46, 3510–3528. 10.1039/C6CS00567E. 28470243

[ref43] Ibáñez M.; Hasler R.; Liu Y.; Dobrozhan O.; Nazarenko O.; Cadavid D.; Cabot A.; Kovalenko M. V. Tuning P-Type Transport in Bottom-Up-Engineered Nanocrystalline Pb Chalcogenides Using Alkali Metal Chalcogenides as Capping Ligands. Chem. Mater. 2017, 29, 7093–7097. 10.1021/acs.chemmater.7b02967. 29434424 PMC5805404

[ref44] Ibáñez M.; Genç A.; Hasler R.; Liu Y.; Dobrozhan O.; Nazarenko O.; De La Mata M.; Arbiol J.; Cabot A.; Kovalenko M. V. Tuning Transport Properties in Thermoelectric Nanocomposites through Inorganic Ligands and Heterostructured Building Blocks. ACS Nano 2019, 13, 6572–6580. 10.1021/acsnano.9b00346. 31185159 PMC6595432

[ref45] Hendricks M. P.; Campos M. P.; Cleveland G. T.; Jen-La Plante I.; Owen J. S. A Tunable Library of Substituted Thiourea Precursors to Metal Sulfide Nanocrystals. Science 2015, 348, 1226–1230. 10.1126/science.aaa2951. 26068846

[ref46] Campos M. P.; Hendricks M. P.; Beecher A. N.; Walravens W.; Swain R. A.; Cleveland G. T.; Hens Z.; Sfeir M. Y.; Owen J. S. A Library of Selenourea Precursors to PbSe Nanocrystals with Size Distributions near the Homogeneous Limit. J. Am. Chem. Soc. 2017, 139, 2296–2305. 10.1021/jacs.6b11021. 28103035

[ref47] Dirmyer M. R.; Martin J.; Nolas G. S.; Sen A.; Badding J. V. Thermal and Electrical Conductivity of Size-Tuned Bismuth Telluride Nanoparticles. Small 2009, 5, 933–937. 10.1002/smll.200801206. 19235804

[ref48] Purkayastha A.; Kim S.; Gandhi D. D.; Ganesan P. G.; Borca-Tasciuc T.; Ramanath G. Molecularly Protected Bismuth Telluride Nanoparticles: Microemulsion Synthesis and Thermoelectric Transport Properties. Adv. Mater. 2006, 18, 2958–2963. 10.1002/adma.200600495.

[ref49] Calcabrini M.; Genç A.; Liu Y.; Kleinhanns T.; Lee S.; Dirin D. N.; Akkerman Q. A.; Kovalenko M. V.; Arbiol J.; Ibáñez M. Exploiting the Lability of Metal Halide Perovskites for Doping Semiconductor Nanocomposites. ACS Energy Lett. 2021, 6, 581–587. 10.1021/acsenergylett.0c02448. 33614964 PMC7887873

[ref50] Li M.; Liu Y.; Zhang Y.; Zuo Y.; Li J.; Lim K. H.; Cadavid D.; Ng K. M.; Cabot A. Crystallographically Textured SnSe Nanomaterials Produced from the Liquid Phase Sintering of Nanocrystals. Dalt. Trans. 2019, 48, 3641–3647. 10.1039/C8DT04414G. 30758366

[ref51] Snyder G. J.; Snyder A. H.; Wood M.; Gurunathan R.; Snyder B. H.; Niu C. Weighted Mobility. Adv. Mater. 2020, 32, 2001537 10.1002/adma.202001537. 32410214

[ref52] Zhang J.; Zhang H.; Cao W.; Pang Z.; Li J.; Shu Y.; Zhu C.; Kong X.; Wang L.; Peng X. Identification of Facet-Dependent Coordination Structures of Carboxylate Ligands on CdSe Nanocrystals. J. Am. Chem. Soc. 2019, 141, 15675–15683. 10.1021/jacs.9b07836. 31503473

[ref53] Choi H.; Ko J. H.; Kim Y. H.; Jeong S. Steric-Hindrance-Driven Shape Transition in PbS Quantum Dots: Understanding Size-Dependent Stability. J. Am. Chem. Soc. 2013, 135, 5278–5281. 10.1021/ja400948t. 23496143

[ref54] Liu L.; Zhuang Z.; Xie T.; Wang Y. G.; Li J.; Peng Q.; Li Y. Shape Control of CdSe Nanocrystals with Zinc Blende Structure. J. Am. Chem. Soc. 2009, 131, 16423–16429. 10.1021/ja903633d. 19902978

[ref55] Hutter E. M.; Bladt E.; Goris B.; Pietra F.; Van Der Bok J. C.; Boneschanscher M. P.; De Mello Donegá C.; Bals S.; Vanmaekelbergh D. Conformal and Atomic Characterization of Ultrathin CdSe Platelets with a Helical Shape. Nano Lett. 2014, 14, 6257–6262. 10.1021/nl5025744. 25347528 PMC4425435

[ref56] Moreels I.; Lambert K.; Smeets D.; De Muynck D.; Nollet T.; Martins J. C.; Vanhaecke F.; Vantomme A.; Delerue C.; Allan G.; et al. Size-Dependent Optical Properties of Colloidal PbS Quantum Dots. ACS Nano 2009, 3, 3023–3030. 10.1021/nn900863a. 19780530

[ref57] Moreels I.; Lambert K.; De Muynck D.; Vanhaecke F.; Poelman D.; Martins J. C.; Allan G.; Hens Z. Composition and Size-Dependent Extinction Coefficient of Colloidal PbSe Quantum Dots. Chem. Mater. 2007, 19, 6101–6106. 10.1021/cm071410q.

[ref58] Mi J. L.; Jensen K. M. Ø.; Tyrsted C.; Bremholm M.; Iversen B. B. In Situ Total X-Ray Scattering Study of the Formation Mechanism and Structural Defects in Anatase TiO_2_ Nanoparticles under Hydrothermal Conditions. CrystEngComm 2015, 17, 6868–6877. 10.1039/C5CE00544B.

[ref59] Boles M. A.; Engel M.; Talapin D. V. Self-Assembly of Colloidal Nanocrystals: From Intricate Structures to Functional Materials. Chem. Rev. 2016, 116, 11220–11289. 10.1021/acs.chemrev.6b00196. 27552640

[ref60] Bao D.; Chen J.; Yu Y.; Liu W.; Huang L.; Han G.; Tang J.; Zhou D.; Yang L.; Chen Z. G. Texture-Dependent Thermoelectric Properties of Nano-Structured Bi_2_Te_3_. Chem. Eng. J. 2020, 388, 124295 10.1016/j.cej.2020.124295.

[ref61] Kim D. H.; Kim C.; Heo S. H.; Kim H. Influence of Powder Morphology on Thermoelectric Anisotropy of Spark-Plasma-Sintered Bi–Te-Based Thermoelectric Materials. Acta Mater. 2011, 59, 405–411. 10.1016/j.actamat.2010.09.054.

[ref62] Han L.; Van Nong N.; Zhang W.; Hung L. T.; Holgate T.; Tashiro K.; Ohtaki M.; Pryds N.; Linderoth S. Effects of Morphology on the Thermoelectric Properties of Al-Doped ZnO. RSC Adv. 2014, 4, 12353–12361. 10.1039/c3ra47617k.

[ref63] Liu W. Di; Shi X. L.; Lin Z. J.; Sun Q.; Han G.; Chen Z. G.; Zou J. Morphology and Texture Engineering Enhancing Thermoelectric Performance of Solvothermal Synthesized Ultralarge SnS Microcrystal. ACS Appl. Energy Mater. 2020, 3, 2192–2199. 10.1021/acsaem.0c00068.

[ref64] Soriano R. B.; Wu J.; Kanatzidis M. G. Size as a Parameter to Stabilize New Phases: Rock Salt Phases of Pb_m_Sb_2n_Se_M+3n_. J. Am. Chem. Soc. 2015, 137, 9937–9942. 10.1021/jacs.5b05562. 26181865

[ref65] Ibáñez M.; Zamani R.; Li W.; Cadavid D.; Gorsse S.; Katcho N. A.; Shavel A.; López A. M.; Morante J. R.; Arbiol J.; et al. Crystallographic Control at the Nanoscale to Enhance Functionality: Polytypic Cu_2_GeSe_3_ Nanoparticles as Thermoelectric Materials. Chem. Mater. 2012, 24, 4615–4622. 10.1021/cm303252q.

[ref66] Soriano R. B.; Arachchige I. U.; Malliakas C. D.; Wu J.; Kanatzidis M. G. Nanoscale Stabilization of New Phases in the PbTe-Sb_2_Te_3_ System: Pb_m_Sb_2n_Te_M+3n_ Nanocrystals. J. Am. Chem. Soc. 2013, 135, 768–774. 10.1021/ja309626q. 23272784

[ref67] Norako M. E.; Greaney M. J.; Brutchey R. L. Synthesis and Characterization of Wurtzite-Phase Copper Tin Selenide Nanocrystals. J. Am. Chem. Soc. 2012, 134, 23–26. 10.1021/ja206929s. 22148639

[ref68] Bree G.; Coughlan C.; Geaney H.; Ryan K. M. Investigation into the Selenization Mechanisms of Wurtzite CZTS Nanorods. ACS Appl. Mater. Interfaces 2018, 10, 7117–7125. 10.1021/acsami.7b18711. 29392941

[ref69] Brutchey R. L. Diorganyl Dichalcogenides as Useful Synthons for Colloidal Semiconductor Nanocrystals. Acc. Chem. Res. 2015, 48, 2918–2926. 10.1021/acs.accounts.5b00362. 26545235

[ref70] Lord R. W.; Fanghanel J.; Holder C. F.; Dabo I.; Schaak R. E. Colloidal Nanoparticles of a Metastable Copper Selenide Phase with Near-Infrared Plasmon Resonance. Chem. Mater. 2020, 32, 10227–10234. 10.1021/acs.chemmater.0c04058.

[ref71] Tappan B. A.; Barim G.; Kwok J. C.; Brutchey R. L. Utilizing Diselenide Precursors toward Rationally Controlled Synthesis of Metastable CuInSe_2_ Nanocrystals. Chem. Mater. 2018, 30, 5704–5713. 10.1021/acs.chemmater.8b02205.

[ref72] Hernández-Pagán E. A.; Robinson E. H.; La Croix A. D.; MacDonald J. E. Direct Synthesis of Novel Cu_2-x_Se Wurtzite Phase. Chem. Mater. 2019, 31, 4619–4624. 10.1021/acs.chemmater.9b02019.

[ref73] Ibáñez M.; Cadavid D.; Zamani R.; García-Castelló N.; Izquierdo-Roca V.; Li W.; Fairbrother A.; Prades J. D.; Shavel A.; Arbiol J.; et al. Composition Control and Thermoelectric Properties of Quaternary Chalcogenide Nanocrystals: The Case of Stannite Cu_2_CdSnSe_4_. Chem. Mater. 2012, 24, 562–570. 10.1021/cm2031812.

[ref74] Ibáñez M.; Zamani R.; Li W.; Shavel A.; Arbiol J.; Morante J. R.; Cabot A. Extending the Nanocrystal Synthesis Control to Quaternary Compositions. Cryst. Growth Des. 2012, 12, 1085–1090. 10.1021/cg201709c.

[ref75] Dwivedi P.; Miyata M.; Higashimine K.; Takahashi M.; Ohta M.; Kubota K.; Takida H.; Akatsuka T.; Maenosono S. Nanobulk Thermoelectric Materials Fabricated from Chemically Synthesized Cu_3_Zn_1-x_Al_x_SnS_5-y_ Nanocrystals. ACS Omega 2019, 4, 16402–16408. 10.1021/acsomega.9b01944. 31616818 PMC6787884

[ref76] Coughlan C.; Guo Y.; Singh S.; Nakahara S.; Ryan K. M. Synthesis of Curved CuIn_1-x_Ga_x_(S_1-y_Se_y_)_2_ Nanocrystals and Complete Characterization of Their Diffraction Contrast Effects. Chem. Mater. 2018, 30, 8679–8689. 10.1021/acs.chemmater.8b04082.

[ref77] Figuerola A.; van Huis M.; Zanella M.; Genovese A.; Marras S.; Falqui A.; Zandbergen H. W.; Cingolani R.; Manna L. Epitaxial CdSe-Au Nanocrystal Heterostructures by Thermal Annealing. Nano Lett. 2010, 10, 3028–3036. 10.1021/nl101482q. 20698616

[ref78] Fantechi E.; Roca A. G.; Sepúlveda B.; Torruella P.; Estradé S.; Peiró F.; Coy E.; Jurga S.; Bastús N. G.; Nogués J.; et al. Seeded Growth Synthesis of Au–Fe_3_O_4_ Heterostructured Nanocrystals: Rational Design and Mechanistic Insights. Chem. Mater. 2017, 29, 4022–4035. 10.1021/acs.chemmater.7b00608.

[ref79] Jeon K. W.; Lee D. G.; Kim Y. K.; Baek K.; Kim K.; Jin T.; Shim J. H.; Park J. Y.; Lee I. S. Mechanistic Insight into the Conversion Chemistry between Au-CuO Heterostructured Nanocrystals Confined inside SiO_2_ Nanospheres. Chem. Mater. 2017, 29, 1788–1795. 10.1021/acs.chemmater.6b05380.

[ref80] Ibáñez M.; Zamani R.; Gorsse S.; Fan J.; Ortega S.; Cadavid D.; Morante J. R.; Arbiol J.; Cabot A. Core-Shell Nanoparticles as Building Blocks for the Bottom-up Production of Functional Nanocomposites: PbTe-PbS Thermoelectric Properties. ACS Nano 2013, 7, 2573–2586. 10.1021/nn305971v. 23448184

[ref81] Scheele M.; Oeschler N.; Veremchuk I.; Peters S. O.; Littig A.; Kornowski A.; Klinke C.; Weller H. Thermoelectric Properties of Lead Chalcogenide Core-Shell Nanostructures. ACS Nano 2011, 5, 8541–8551. 10.1021/nn2017183. 21981245

[ref82] Buonsanti R.; Loiudice A.; Mantella V. Colloidal Nanocrystals as Precursors and Intermediates in Solid State Reactions for Multinary Oxide Nanomaterials. Acc. Chem. Res. 2021, 54, 754–764. 10.1021/acs.accounts.0c00698. 33492926

[ref83] Hartley C. L.; Kessler M. L.; Dempsey J. L. Molecular-Level Insight into Semiconductor Nanocrystal Surfaces. J. Am. Chem. Soc. 2021, 143, 1251–1266. 10.1021/jacs.0c10658. 33442974

[ref84] Bederak D.; Dirin D. N.; Sukharevska N.; Momand J.; Kovalenko M. V.; Loi M. A. S-Rich PbS Quantum Dots: A Promising p-Type Material for Optoelectronic Devices. Chem. Mater. 2021, 33, 320–326. 10.1021/acs.chemmater.0c03865.

[ref85] Moreels I.; Fritzinger B.; Martins J. C.; Hens Z. Surface Chemistry of Colloidal PbSe Nanocrystals. J. Am. Chem. Soc. 2008, 130, 15081–15086. 10.1021/ja803994m. 18928251

[ref86] Liu Y.; Calcabrini M.; Yu Y.; Genç A.; Chang C.; Costanzo T.; Kleinhanns T.; Lee S.; Llorca J.; Cojocaru-Mirédin O.; et al. The Importance of Surface Adsorbates in Solution-Processed Thermoelectric Materials: The Case of SnSe. Adv. Mater. 2021, 33, 2106858 10.1002/adma.202106858. PMC11468704 34626034

[ref87] Mohapatra P.; Shaw S.; Mendivelso-Perez D.; Bobbitt J. M.; Silva T. F.; Naab F.; Yuan B.; Tian X.; Smith E. A.; Cademartiri L. Calcination Does Not Remove All Carbon from Colloidal Nanocrystal Assemblies. Nat. Commun. 2017, 8, 2038 10.1038/s41467-017-02267-9. 29229916 PMC5725572

[ref88] Cargnello M.; Chen C.; Diroll B. T.; Doan-Nguyen V. V. T.; Gorte R. J.; Murray C. B. Efficient Removal of Organic Ligands from Supported Nanocrystals by Fast Thermal Annealing Enables Catalytic Studies on Well-Defined Active Phases. J. Am. Chem. Soc. 2015, 137, 6906–6911. 10.1021/jacs.5b03333. 25961673

[ref89] Ibáñez M.; Korkosz R. J.; Luo Z.; Riba P.; Cadavid D.; Ortega S.; Cabot A.; Kanatzidis M. G. Electron Doping in Bottom-up Engineered Thermoelectric Nanomaterials through HCl-Mediated Ligand Displacement. J. Am. Chem. Soc. 2015, 137, 4046–4049. 10.1021/jacs.5b00091. 25762361

[ref90] Kovalenko M. V.; Spokoyny B.; Lee J. S.; Scheele M.; Weber A.; Perera S.; Landry D.; Talapin D. V. Semiconductor Nanocrystals Functionalized with Antimony Telluride Zintl Ions for Nanostructured Thermoelectrics. J. Am. Chem. Soc. 2010, 132, 6686–6695. 10.1021/ja909591x. 20423085

[ref91] Dai M. Q.; Yung L. Y. L. Ethylenediamine-Assisted Ligand Exchange and Phase Transfer of Oleophilic Quantum Dots: Stripping of Original Ligands and Preservation of Photoluminescence. Chem. Mater. 2013, 25, 2193–2201. 10.1021/cm304136a.

[ref92] Kumar A. P.; Huy B. T.; Kumar B. P.; Kim J. H.; Dao V. D.; Choi H. S.; Lee Y. I. Novel Dithiols as Capping Ligands for CdSe Quantum Dots: Optical Properties and Solar Cell Applications. J. Mater. Chem. C 2015, 3, 1957–1964. 10.1039/C4TC01863J.

[ref93] Lokteva I.; Radychev N.; Witt F.; Borchert H.; Parisi J.; Kolny-Olesiak J. Surface Treatment of Cdse Nanoparticles for Application in Hybrid Solar Cells: The Effect of Multiple Ligand Exchange with Pyridine. J. Phys. Chem. C 2010, 114, 12784–12791. 10.1021/jp103300v.

[ref94] Boles M. A.; Ling D.; Hyeon T.; Talapin D. V. The Surface Science of Nanocrystals. Nat. Mater. 2016, 15, 141–153. 10.1038/nmat4526. 26796733

[ref95] Sharma R.; Sawvel A. M.; Barton B.; Dong A.; Buonsanti R.; Llordes A.; Schaible E.; Axnanda S.; Liu Z.; Urban J. J.; et al. Nanocrystal Superlattice Embedded within an Inorganic Semiconducting Matrix by in Situ Ligand Exchange: Fabrication and Morphology. Chem. Mater. 2015, 27, 2755–2758. 10.1021/cm504716s.

[ref96] Jiang C.; Lee J. S.; Talapin D. V. Soluble Precursors for CuInSe_2_, CuIn_1-x_Ga_x_Se_2_, and Cu_2_ZnSn(S,Se)_4_ Based on Colloidal Nanocrystals and Molecular Metal Chalcogenide Surface Ligands. J. Am. Chem. Soc. 2012, 134, 5010–5013. 10.1021/ja2105812. 22329720

[ref97] Dolzhnikov D. S.; Zhang H.; Jang J.; Son J. S.; Panthani M. G.; Shibata T.; Chattopadhyay S.; Talapin D. V. Composition-Matched Molecular “Solders” for Semiconductors. Science 2015, 347, 425–428. 10.1126/science.1260501. 25569110

[ref98] Liu Y.; Calcabrini M.; Yu Y.; Lee S.; Chang C.; David J.; Ghosh T.; Spadaro M. C.; Xie C.; Cojocaru-Mirédin O.; et al. Defect Engineering in Solution-Processed Polycrystalline SnSe Leads to High Thermoelectric Performance. ACS Nano 2022, 16, 78–88. 10.1021/acsnano.1c06720. PMC8793148 34549956

[ref99] Carrete A.; Shavel A.; Fontané X.; Montserrat J.; Fan J.; Ibáñez M.; Saucedo E.; Pérez-Rodríguez A.; Cabot A. Antimony-Based Ligand Exchange to Promote Crystallization in Spray-Deposited Cu_2_ZnSnSe_4_ Solar Cells. J. Am. Chem. Soc. 2013, 135, 15982–15985. 10.1021/ja4068639. 24116944

[ref100] Chang C.; Ibáñez M. Enhanced Thermoelectric Performance by Surface Engineering in SnTe-PbS Nanocomposites. Materials 2021, 14, 5416 10.3390/ma14185416. 34576640 PMC8466123

[ref101] Buckley J. J.; Greaney M. J.; Brutchey R. L. Ligand Exchange of Colloidal Cdse Nanocrystals with Stibanates Derived from Sb_2_S_3_ Dissolved in a Thiol-Amine Mixture. Chem. Mater. 2014, 26, 6311–6317. 10.1021/cm503324k.

[ref102] Ibañez M.; Hasler R.; Genc A.; Liu Y.; Kuster B.; Schuster M.; Dobrozhan O.; Cadavid D.; Arbiol J.; Cabot A.; et al. Ligand-Mediated Band Engineering in Bottom-up Assembled SnTe Nanocomposites for Thermoelectric Energy Conversion. J. Am. Chem. Soc. 2019, 141, 8025–8029. 10.1021/jacs.9b01394. 31017419 PMC6588270

[ref103] Panthani M. G.; Kurley J. M.; Crisp R. W.; Dietz T. C.; Ezzyat T.; Luther J. M.; Talapin D. V. High Efficiency Solution Processed Sintered CdTe Nanocrystal Solar Cells: The Role of Interfaces. Nano Lett. 2014, 14, 670–675. 10.1021/nl403912w. 24364381

[ref104] Zhang H.; Jang J.; Liu W.; Talapin D. V. Colloidal Nanocrystals with Inorganic Halide, Pseudohalide, and Halometallate Ligands. ACS Nano 2014, 8, 7359–7369. 10.1021/nn502470v. 24988140

[ref105] Huang J.; Liu W.; Dolzhnikov D. S.; Protesescu L.; Kovalenko M. V.; Koo B.; Chattopadhyay S.; Shenchenko E. V.; Talapin D. V. Surface Functionalization of Semiconductor and Oxide Nanocrystals with Small Inorganic Oxoanions (PO_4_^3-^, MoO_4_^2-^) and Polyoxometalate Ligands. ACS Nano 2014, 8, 9388–9402. 10.1021/nn503458y. 25181260

[ref106] Dirin D. N.; Dreyfuss S.; Bodnarchuk M. I.; Nedelcu G.; Papagiorgis P.; Itskos G.; Kovalenko M. V. Lead Halide Perovskites and Other Metal Halide Complexes as Inorganic Capping Ligands for Colloidal Nanocrystals. J. Am. Chem. Soc. 2014, 136, 6550–6553. 10.1021/ja5006288. 24746226 PMC4524702

[ref107] Fafarman A. T.; Koh W. K.; Diroll B. T.; Kim D. K.; Ko D. K.; Oh S. J.; Ye X.; Doan-Nguyen V.; Crump M. R.; Reifsnyder D. C.; et al. Thiocyanate-Capped Nanocrystal Colloids: Vibrational Reporter of Surface Chemistry and Solution-Based Route to Enhanced Coupling in Nanocrystal Solids. J. Am. Chem. Soc. 2011, 133, 15753–15761. 10.1021/ja206303g. 21848336

[ref108] Nag A.; Kovalenko M. V.; Lee J. S.; Liu W.; Spokoyny B.; Talapin D. V. Tetragonal - Orthorhombic - Cubic Phase Transitions in Ag_2_Se Nanocrystals. J. Am. Chem. Soc. 2011, 133, 10612–10620. 10.1021/ja2029415. 21682249

[ref109] Oh S. J.; Straus D. B.; Zhao T.; Choi J. H.; Lee S. W.; Gaulding E. A.; Murray C. B.; Kagan C. R. Engineering the Surface Chemistry of Lead Chalcogenide Nanocrystal Solids to Enhance Carrier Mobility and Lifetime in Optoelectronic Devices. Chem. Commun. 2017, 53, 728–731. 10.1039/C6CC07916D. 27990537

[ref110] Balazs D. M.; Dirin D. N.; Fang H. H.; Protesescu L.; Ten Brink G. H.; Kooi B. J.; Kovalenko M. V.; Loi M. A. Counterion-Mediated Ligand Exchange for PbS Colloidal Quantum Dot Superlattices. ACS Nano 2015, 9, 11951–11959. 10.1021/acsnano.5b04547. 26512884 PMC4690194

[ref111] Bederak D.; Balazs D. M.; Sukharevska N. V.; Shulga A. G.; Abdu-Aguye M.; Dirin D. N.; Kovalenko M. V.; Loi M. A. Comparing Halide Ligands in PbS Colloidal Quantum Dots for Field-Effect Transistors and Solar Cells. ACS Appl. Nano Mater. 2018, 1, 6882–6889. 10.1021/acsanm.8b01696. 30613830 PMC6317010

[ref112] Chang C.; Liu Y.; Lee S. H.; Spadaro M. C.; Koskela K. M.; Kleinhanns T.; Costanzo T.; Arbiol J.; Brutchey R. L.; Ibáñez M. Surface Functionalization of Surfactant-Free Particles: A Strategy to Tailor the Properties of Nanocomposites for Enhanced Thermoelectric Performance. Angew. Chem. 2022, 61, e202207002 10.1002/anie.202207002. 35799379 PMC9542085

[ref113] Schrader I.; Warneke J.; Neumann S.; Grotheer S.; Swane A. A.; Kirkensgaard J. J. K.; Arenz M.; Kunz S. Surface Chemistry of “Unprotected” Nanoparticles: A Spectroscopic Investigation on Colloidal Particles. J. Phys. Chem. C 2015, 119, 17655–17661. 10.1021/acs.jpcc.5b03863.

[ref114] Ansar S. M.; Ameer F. S.; Hu W.; Zou S.; Pittman C. U.; Zhang D. Removal of Molecular Adsorbates on Gold Nanoparticles Using Sodium Borohydride in Water. Nano Lett. 2013, 13, 1226–1229. 10.1021/nl304703w. 23387414

[ref115] Nelson A.; Zong Y.; Fritz K. E.; Suntivich J.; Robinson R. D. Assessment of Soft Ligand Removal Strategies: Alkylation as a Promising Alternative to High-Temperature Treatments for Colloidal Nanoparticle Surfaces. ACS Mater. Lett. 2019, 1, 177–184. 10.1021/acsmaterialslett.9b00089.

[ref116] Rosen E. L.; Buonsanti R.; Llordes A.; Sawvel A. M.; Milliron D. J.; Helms B. A. Exceptionally Mild Reactive Stripping of Native Ligands from Nanocrystal Surfaces by Using Meerwein’s Salt. Angew. Chemie Int. Ed. 2012, 51, 684–689. 10.1002/anie.201105996. 22147424

[ref117] Duong J. T.; Bailey M. J.; Pick T. E.; McBride P. M.; Rosen E. L.; Buonsanti R.; Milliron D. J.; Helms B. A. Efficient Polymer Passivation of Ligand-Stripped Nanocrystal Surfaces. J. Polym. Sci. Part A Polym. Chem. 2012, 50, 3719–3727. 10.1002/pola.26178.

[ref118] Gadiyar C.; Loiudice A.; D’Ambra F.; Oveisi E.; Stoian D.; Iyengar P.; Castilla-Amorós L.; Mantella V.; Buonsanti R. Nanocrystals as Precursors in Solid-State Reactions for Size- and Shape-Controlled Polyelemental Nanomaterials. J. Am. Chem. Soc. 2020, 142, 15931–15940. 10.1021/jacs.0c06556. 32845630

[ref119] Mainz R.; Singh A.; Levcenko S.; Klaus M.; Genzel C.; Ryan K. M.; Unold T. Phase-Transition-Driven Growth of Compound Semiconductor Crystals from Ordered Metastable Nanorods. Nat. Commun. 2014, 5, 3133 10.1038/ncomms4133. 24448477

[ref120] Santos P. J.; Gabrys P. A.; Zornberg L. Z.; Lee M. S.; Macfarlane R. J. Macroscopic Materials Assembled from Nanoparticle Superlattices. Nature 2021, 591, 586–591. 10.1038/s41586-021-03355-z. 33762767

[ref121] Biswas K.; He J.; Blum I. D.; Wu C. I.; Hogan T. P.; Seidman D. N.; Dravid V. P.; Kanatzidis M. G. High-Performance Bulk Thermoelectrics with All-Scale Hierarchical Architectures. Nature 2012, 489, 414–418. 10.1038/nature11439. 22996556

[ref122] Qin Y.; Xiao Y.; Zhao L. D. Carrier Mobility Does Matter for Enhancing Thermoelectric Performance. APL Mater. 2020, 8, 010901 10.1063/1.5144097.

[ref123] Cadavid D.; Ibáñez M.; Gorsse S.; López A. M.; Cirera A.; Morante J. R.; Cabot A. Bottom-up Processing of Thermoelectric Nanocomposites from Colloidal Nanocrystal Building Blocks: The Case of Ag_2_Te–PbTe. J. Nanoparticle Res. 2012, 14, 1328 10.1007/s11051-012-1328-0.

[ref124] Zhang Y.; Xing C.; Liu Y.; Spadaro M. C.; Wang X.; Li M.; Xiao K.; Zhang T.; Guardia P.; Lim K. H.; et al. Doping-Mediated Stabilization of Copper Vacancies to Promote Thermoelectric Properties of Cu_2–x_S. Nano Energy 2021, 85, 105991 10.1016/j.nanoen.2021.105991.

[ref125] Liu Y.; Cadavid D.; Ibáñez M.; Ortega S.; Martí-Sánchez S.; Dobrozhan O.; Kovalenko M. V.; Arbiol J.; Cabot A. Thermoelectric Properties of Semiconductor-Metal Composites Produced by Particle Blending. APL Mater. 2016, 4, 104813 10.1063/1.4961679.

[ref126] Chu Z.; Han Y.; Kral P.; Klajn R. "Precipitation on Nanoparticles": Attractive Intermolecular Interactions Stabilize Specific Ligand Ratios on the Surfaces of Nanoparticles. Angew. Chemie Int. Ed. 2018, 57, 7023–7027. 10.1002/anie.201800673. 29673022

[ref127] Zhao H.; Sen S.; Udayabhaskararao T.; Sawczyk M.; Kucanda K.; Manna D.; Kundu P. K.; Lee J. W.; Král P.; Klajn R. Reversible Trapping and Reaction Acceleration within Dynamically Self-Assembling Nanoflasks. Nat. Nanotechnol. 2016, 11, 82–88. 10.1038/nnano.2015.256. 26595335

[ref128] Coropceanu I.; Janke E. M.; Portner J.; Haubold D.; Nguyen T. D.; Das A.; Tanner C. P. N.; Utterback J. K.; Teitelbaum S. W.; Hudson M. H.; et al. Self-Assembly of Nanocrystals into Strongly Electronically Coupled All-Inorganic Supercrystals. Science 2022, 375, 1422–1426. 10.1126/science.abm6753. 35324292

[ref129] Cherniukh I.; Rainò G.; Stöferle T.; Burian M.; Travesset A.; Naumenko D.; Amenitsch H.; Erni R.; Mahrt R. F.; Bodnarchuk M. I.; et al. Perovskite-Type Superlattices from Lead Halide Perovskite Nanocubes. Nature 2021, 593, 535–542. 10.1038/s41586-021-03492-5. 34040208

[ref130] Salzmann B. B. V.; Van Der Sluijs M. M.; Soligno G.; Vanmaekelbergh D. Oriented Attachment: From Natural Crystal Growth to a Materials Engineering Tool. Acc. Chem. Res. 2021, 54, 787–797. 10.1021/acs.accounts.0c00739. 33502844 PMC7893701

[ref131] Harman T. C.; Taylor P. J.; Walsh M. P.; LaForge B. E. Quantum Dot Superlattice Thermoelectric Materials and Devices. Science 2002, 297, 2229–2232. 10.1126/science.1072886. 12351781

[ref132] Venkatasubramanian R.; Siivola E.; Colpitts T.; O’Quinn B. Thin-Film Thermoelectric Devices with High Room-Temperature Figures of Merit. Nature 2001, 413, 597–602. 10.1038/35098012. 11595940

[ref133] Shi X. L.; Liu W. Di; Wu A. Y.; Nguyen V. T.; Gao H.; Sun Q.; Moshwan R.; Zou J.; Chen Z. G. Optimization of Sodium Hydroxide for Securing High Thermoelectric Performance in Polycrystalline Sn_1–x_Se via Anisotropy and Vacancy Synergy. InfoMat 2020, 2, 1201–1215. 10.1002/inf2.12057.

[ref134] Nandihalli N.; Gregory D. H.; Mori T. Energy-Saving Pathways for Thermoelectric Nanomaterial Synthesis: Hydrothermal/Solvothermal, Microwave-Assisted, Solution-Based, and Powder Processing. Adv. Sci. 2022, 9, 2106052 10.1002/advs.202106052. PMC9443476 35843868

[ref135] Wei J.; Yang L.; Ma Z.; Song P.; Zhang M.; Ma J.; Yang F.; Wang X. Review of Current High-ZT Thermoelectric Materials. J. Mater. Sci. 2020, 55, 12642–12704. 10.1007/s10853-020-04949-0.

[ref136] Kim F.; Kwon B.; Eom Y.; Lee J. E.; Park S.; Jo S.; Park S. H.; Kim B. S.; Im H. J.; Lee M. H.; et al. 3D Printing of Shape-Conformable Thermoelectric Materials Using All-Inorganic Bi_2_Te_3_-Based Inks. Nat. Energy 2018, 3, 301–309. 10.1038/s41560-017-0071-2.

[ref137] Yan Y.; Geng W.; Qiu J.; Ke H.; Luo C.; Yang J.; Uher C.; Tang X. Thermoelectric Properties of N-Type ZrNiSn Prepared by Rapid Non-Equilibrium Laser Processing. RSC Adv. 2018, 8, 15796–15803. 10.1039/C8RA00992A. 35539494 PMC9080093

[ref138] Zeng M.; Zavanelli D.; Chen J.; Saeidi-Javash M.; Du Y.; Leblanc S.; Snyder G. J.; Zhang Y. Printing Thermoelectric Inks toward Next-Generation Energy and Thermal Devices. Chem. Soc. Rev. 2022, 51, 485–512. 10.1039/D1CS00490E. 34761784

[ref139] Eom Y.; Kim F.; Yang S. E.; Son J. S.; Chae H. G. Rheological Design of 3D Printable All-Inorganic Inks Using BiSbTe-Based Thermoelectric Materials. J. Rheol. 2019, 63, 291 10.1122/1.5058078.

[ref140] Burton M. R.; Mehraban S.; Beynon D.; McGettrick J.; Watson T.; Lavery N. P.; Carnie M. J. 3D Printed SnSe Thermoelectric Generators with High Figure of Merit. Adv. Energy Mater. 2019, 9, 1900201 10.1002/aenm.201900201.

[ref141] Albrecht W.; Bals S. Fast Electron Tomography for Nanomaterials. J. Phys. Chem. C 2020, 124, 27276–27286. 10.1021/acs.jpcc.0c08939.

[ref142] Yu P.; Beard M. C.; Ellingson R. J.; Ferrere S.; Curtis C.; Drexler J.; Luiszer F.; Nozik A. J. Absorption Cross-Section and Related Optical Properties of Colloidal InAs Quantum Dots. J. Phys. Chem. B 2005, 109, 7084–7087. 10.1021/jp046127i. 16851806

[ref143] Cademartiri L.; Montanari E.; Calestani G.; Migliori A.; Guagliardi A.; Ozin G. A. Size-Dependent Extinction Coefficients of PbS Quantum Dots. J. Am. Chem. Soc. 2006, 128, 10337–10346. 10.1021/ja063166u. 16881666

[ref144] Ding D.; Lu C.; Tang Z. Bottom Up Chalcogenide Thermoelectric Materials from Solution-Processed Nanostructures. Adv. Mater. Interfaces 2017, 4, 1700517 10.1002/admi.201700517.

[ref145] Ramos A. P. Dynamic Light Scattering Applied to Nanoparticle Characterization. In Nanocharacterization Techniques; Da Ráz A. L., Ferreira M., Leite F. d. L., Oliveria O. N. Jr., Eds.; Elsevier, 2017; pp 99–110.

[ref146] Hens Z.; De Roo J. Atomically Precise Nanocrystals. J. Am. Chem. Soc. 2020, 142, 15627–15637. 10.1021/jacs.0c05082. 32804488

[ref147] Zhang G.; Wang W.; Li X. Enhanced Thermoelectric Properties of Core/Shell Heterostructure Nanowire Composites. Adv. Mater. 2008, 20, 3654–3656. 10.1002/adma.200800162.

[ref148] Zhang G.; Fang H.; Yang H.; Jauregui L. A.; Chen Y. P.; Wu Y. Design Principle of Telluride-Based Nanowire Heterostructures for Potential Thermoelectric Applications. Nano Lett. 2012, 12, 3627–3633. 10.1021/nl301327d. 22731993

[ref149] De La Mata M.; Magen C.; Gazquez J.; Utama M. I. B.; Heiss M.; Lopatin S.; Furtmayr F.; Fernández-Rojas C. J.; Peng B.; Morante J. R.; et al. Polarity Assignment in ZnTe, GaAs, ZnO, and GaN-AlN Nanowires from Direct Dumbbell Analysis. Nano Lett. 2012, 12, 2579–2586. 10.1021/nl300840q. 22493937

[ref150] Li C.; Zhang Q.; Mayoral A. Ten Years of Aberration Corrected Electron Microscopy for Ordered Nanoporous Materials. ChemCatChem. 2020, 12, 1248–1269. 10.1002/cctc.201901861.

[ref151] Li T.; Senesi A. J.; Lee B. Small Angle X-Ray Scattering for Nanoparticle Research. Chem. Rev. 2016, 116, 11128–11180. 10.1021/acs.chemrev.5b00690. 27054962

[ref152] Anand S.; Wolverton C.; Snyder G. J. Thermodynamic Guidelines for Maximum Solubility. Chem. Mater. 2022, 34, 1638–1648. 10.1021/acs.chemmater.1c03715.

[ref153] Tang Y.; Chen S.-w.; Snyder G. J. Temperature Dependent Solubility of Yb in Yb–CoSb_3_ Skutterudite and Its Effect on Preparation, Optimization and Lifetime of Thermoelectrics. J. Mater. 2015, 1, 75–84. 10.1016/j.jmat.2015.03.008.

[ref154] Frenkel A. I. Applications of Extended X-Ray Absorption Fine-Structure Spectroscopy to Studies of Bimetallic Nanoparticle Catalysts. Chem. Soc. Rev. 2012, 41, 8163–8178. 10.1039/c2cs35174a. 22833100

[ref155] Tarachand; Sharma V.; Bhatt R.; Ganesan V.; Okram G. S. A Catalyst-Free New Polyol Method Synthesized Hot-Pressed Cu-Doped Bi2S3 Nanorods and Their Thermoelectric Properties. Nano Res. 2016, 9, 3291–3304. 10.1007/s12274-016-1207-6.

[ref156] Li M.; Liu Y.; Zhang Y.; Han X.; Zhang T.; Zuo Y.; Xie C.; Xiao K.; Arbiol J.; Llorca J.; et al. Effect of the Annealing Atmosphere on Crystal Phase and Thermoelectric Properties of Copper Sulfide. ACS Nano 2021, 15, 4967–4978. 10.1021/acsnano.0c09866. 33645986

[ref157] Li M.; Zhang Y.; Zhang T.; Zuo Y.; Xiao K.; Arbiol J.; Llorca J.; Liu Y.; Cabot A. Enhanced Thermoelectric Performance of N-Type Bi_2_Se_3_ Nanosheets through Sn Doping. Nanomaterials 2021, 11, 1827 10.3390/nano11071827. 34361214 PMC8308202

[ref158] Peters J. L.; Van Den Bos K. H. W.; Van Aert S.; Goris B.; Bals S.; Vanmaekelbergh D. Ligand-Induced Shape Transformation of PbSe Nanocrystals. Chem. Mater. 2017, 29, 4122–4128. 10.1021/acs.chemmater.7b01103. 28503030 PMC5425942

[ref159] Deblock L.; Goossens E.; Pokratath R.; De Buysser K.; De Roo J. Mapping out the Aqueous Surface Chemistry of Metal Oxide Nanocrystals: Carboxylate, Phosphonate, and Catecholate Ligands. JACS Au 2022, 2, 711–722. 10.1021/jacsau.1c00565. 35373200 PMC8969999

[ref160] Oliva-Puigdomènech A.; De Roo J.; Kuhs J.; Detavernier C.; Martins J. C.; Hens Z. Ligand Binding to Copper Nanocrystals: Amines and Carboxylic Acids and the Role of Surface Oxides. Chem. Mater. 2019, 31, 2058–2067. 10.1021/acs.chemmater.8b05107.

[ref161] Hens Z.; Martins J. C. A Solution NMR Toolbox for Characterizing the Surface Chemistry of Colloidal Nanocrystals. Chem. Mater. 2013, 25, 1211–1221. 10.1021/cm303361s.

[ref162] Chen Y.; Dorn R. W.; Hanrahan M. P.; Wei L.; Blome-Fernández R.; Medina-Gonzalez A. M.; Adamson M. A. S.; Flintgruber A. H.; Vela J.; Rossini A. J. Revealing the Surface Structure of CdSe Nanocrystals by Dynamic Nuclear Polarization-Enhanced ^77^Se and ^113^Cd Solid-State NMR Spectroscopy. J. Am. Chem. Soc. 2021, 143, 8747–8760. 10.1021/jacs.1c03162. 34085812

[ref163] Zherebetskyy D.; Scheele M.; Zhang Y.; Bronstein N.; Thompson C.; Britt D.; Salmeron M.; Alivisatos P.; Wang L. W. Hydroxylation of the Surface of PbS Nanocrystals Passivated with Oleic Acid. Science 2014, 344, 1380–1384. 10.1126/science.1252727. 24876347

[ref164] De Roo J.; Van Den Broeck F.; De Keukeleere K.; Martins J. C.; Van Driessche I.; Hens Z. Unravelling the Surface Chemistry of Metal Oxide Nanocrystals, the Role of Acids and Bases. J. Am. Chem. Soc. 2014, 136, 9650–9657. 10.1021/ja5032979. 24945901

[ref165] Green P. B.; Villanueva F. Y.; Demmans K. Z.; Imperiale C. J.; Hasham M.; Nikbin E.; Howe J. Y.; Burns D. C.; Wilson M. W. B. PbS Nanocrystals Made Using Excess Lead Chloride Have a Halide-Perovskite-Like Surface. Chem. Mater. 2021, 33, 9270–9284. 10.1021/acs.chemmater.1c02962.

[ref166] Dickson M. J. IUCr. The Significance of Texture Parameters in Phase Analysis by X-Ray Diffraction. J. Appl. Crystallogr. 1969, 2, 176–180. 10.1107/S0021889869006881.

[ref167] Vegard L. Die Konstitution Der Mischkristalle Und Die Raumfüllung Der Atome. Z. Physik 1921, 5, 17–26. 10.1007/BF01349680.

[ref168] Song S. W.; Mao J.; Bordelon M.; He R.; Wang Y. M.; Shuai J.; Sun J. Y.; Lei X. B.; Ren Z. S.; Chen S.; et al. Joint Effect of Magnesium and Yttrium on Enhancing Thermoelectric Properties of N-Type Zintl Mg_3+δ_Y_0.02_Sb_1.5_Bi_0.5_. Mater. Today Phys. 2019, 8, 25–33. 10.1016/j.mtphys.2018.12.004.

[ref169] Christensen S.; Bindzus N.; Sist M.; Takata M.; Iversen B. B. Structural Disorder, Anisotropic Micro-Strain and Cation Vacancies in Thermo-Electric Lead Chalcogenides. Phys. Chem. Chem. Phys. 2016, 18, 15874–15883. 10.1039/C6CP01730D. 27240951

[ref170] Yang X.; Jiang Z.; Li J.; Kang H.; Liu D.; Yang F.; Chen Z.; Guo E.; Jiang X.; Wang T. Identification of the Intrinsic Atomic Disorder in ZrNiSn-Based Alloys and Their Effects on Thermoelectric Properties. Nano Energy 2020, 78, 105372 10.1016/j.nanoen.2020.105372.

[ref171] Van Tendeloo G.; Bals S.; Van Aert S.; Verbeeck J.; Van Dyck D. Advanced Electron Microscopy for Advanced Materials. Adv. Mater. 2012, 24, 5655–5675. 10.1002/adma.201202107. 22907862

[ref172] Sinha S.; Zhu T.; France-Lanord A.; Sheng Y.; Grossman J. C.; Porfyrakis K.; Warner J. H. Atomic Structure and Defect Dynamics of Monolayer Lead Iodide Nanodisks with Epitaxial Alignment on Graphene. Nat. Commun. 2020, 11, 823 10.1038/s41467-020-14481-z. 32041958 PMC7010709

[ref173] Hong J.; Hu Z.; Probert M.; Li K.; Lv D.; Yang X.; Gu L.; Mao N.; Feng Q.; Xie L.; et al. Exploring Atomic Defects in Molybdenum Disulphide Monolayers. Nat. Commun. 2015, 6, 6293 10.1038/ncomms7293. 25695374 PMC4346634

[ref174] Gault B.; Chiaramonti A.; Cojocaru-Mirédin O.; Stender P.; Dubosq R.; Freysoldt C.; Makineni S. K.; Li T.; Moody M.; Cairney J. M. Atom Probe Tomography. Nat. Rev. Methods Prim. 2021, 1, 51 10.1038/s43586-021-00047-w. PMC10502706 37719173

[ref175] Kim Y. J.; Zhao L. D.; Kanatzidis M. G.; Seidman D. N. Analysis of Nanoprecipitates in a Na-Doped PbTe-SrTe Thermoelectric Material with a High Figure of Merit. ACS Appl. Mater. Interfaces 2017, 9, 21791–21797. 10.1021/acsami.7b04098. 28590114

[ref176] Zheng Y.; Slade T. J.; Hu L.; Tan X. Y.; Luo Y.; Luo Z. Z.; Xu J.; Yan Q.; Kanatzidis M. G. Defect Engineering in Thermoelectric Materials: What Have We Learned?. Chem. Soc. Rev. 2021, 50, 9022–9054. 10.1039/D1CS00347J. 34137396

[ref177] Li Z.; Xiao C.; Fan S.; Deng Y.; Zhang W.; Ye B.; Xie Y. Dual Vacancies: An Effective Strategy Realizing Synergistic Optimization of Thermoelectric Property in BiCuSeO. J. Am. Chem. Soc. 2015, 137, 6587–6593. 10.1021/jacs.5b01863. 25927811

[ref178] Tan X.; Lan J. Le; Hu K.; Xu B.; Liu Y.; Zhang P.; Cao X. Z.; Zhu Y.; Xu W.; Lin Y. H.; et al. Boosting the Thermoelectric Performance of Bi_2_O_2_Se by Isovalent Doping. J. Am. Ceram. Soc. 2018, 101, 4634–4644. 10.1111/jace.15720.

[ref179] He H. F.; Li X. F.; Chen Z. Q.; Zheng Y.; Yang D. W.; Tang X. F. Interplay between Point Defects and Thermal Conductivity of Chemically Synthesized Bi_2_Te_3_Nanocrystals Studied by Positron Annihilation. J. Phys. Chem. C 2014, 118, 22389–22394. 10.1021/jp508085a.

[ref180] Chen Z.; Jian Z.; Li W.; Chang Y.; Ge B.; Hanus R.; Yang J.; Chen Y.; Huang M.; Snyder G. J.; et al. Lattice Dislocations Enhancing Thermoelectric PbTe in Addition to Band Convergence. Adv. Mater. 2017, 29, 1606768 10.1002/adma.201606768. 28397364

[ref181] Abdellaoui L.; Chen Z.; Yu Y.; Luo T.; Hanus R.; Schwarz T.; Bueno Villoro R.; Cojocaru-Mirédin O.; Snyder G. J.; Raabe D.; et al. Parallel Dislocation Networks and Cottrell Atmospheres Reduce Thermal Conductivity of PbTe Thermoelectrics. Adv. Funct. Mater. 2021, 31, 2101214 10.1002/adfm.202101214.

[ref182] Yu Y.; Zhang S.; Mio A. M.; Gault B.; Sheskin A.; Scheu C.; Raabe D.; Zu F.; Wuttig M.; Amouyal Y.; et al. Ag-Segregation to Dislocations in PbTe-Based Thermoelectric Materials. ACS Appl. Mater. Interfaces 2018, 10, 3609–3615. 10.1021/acsami.7b17142. 29309116

[ref183] Meng X.; Liu Z.; Cui B.; Qin D.; Geng H.; Cai W.; Fu L.; He J.; Ren Z.; Sui J. Grain Boundary Engineering for Achieving High Thermoelectric Performance in N-Type Skutterudites. Adv. Energy Mater. 2017, 7, 1602582 10.1002/aenm.201602582.

[ref184] Kuo J. J.; Yu Y.; Kang S. D.; Cojocaru-Mirédin O.; Wuttig M.; Snyder G. J. Mg Deficiency in Grain Boundaries of N-Type Mg_3_Sb_2_ Identified by Atom Probe Tomography. Adv. Mater. Interfaces 2019, 6, 1900429 10.1002/admi.201900429.

[ref185] Ozturk C. E.; Ugraskan V.; Yazici O. Thermoelectric Properties of Titanium Carbide Filled Polypyrrole Hybrid Composites. J. Electron. Mater. 2022, 51, 5246–5252. 10.1007/s11664-022-09776-4.

[ref186] Virtudazo R. V. R.; Srinivasan B.; Guo Q.; Wu R.; Takei T.; Shimasaki Y.; Wada H.; Kuroda K.; Bernik S.; Mori T. Improvement in the Thermoelectric Properties of Porous Networked Al-Doped ZnO Nanostructured Materials Synthesized via an Alternative Interfacial Reaction and Low-Pressure SPS Processing. Inorg. Chem. Front. 2020, 7, 4118–4132. 10.1039/D0QI00888E.

[ref187] Geuchies J. J.; Van Overbeek C.; Evers W. H.; Goris B.; De Backer A.; Gantapara A. P.; Rabouw F. T.; Hilhorst J.; Peters J. L.; Konovalov O.; et al. In Situ Study of the Formation Mechanism of Two-Dimensional Superlattices from PbSe Nanocrystals. Nat. Mater. 2016, 15, 1248–1254. 10.1038/nmat4746. 27595349

[ref188] Zheng Q.; Jiang J.; Li X.; Bustillo K. C.; Zheng H. In Situ TEM Observation of Calcium Silicate Hydrate Nanostructure at High Temperatures. Cem. Concr. Res. 2021, 149, 106579 10.1016/j.cemconres.2021.106579.

[ref189] Dargusch M.; Shi X. L.; Tran X. Q.; Feng T.; Somidin F.; Tan X.; Liu W.; Jack K.; Venezuela J.; Maeno H.; et al. In-Situ Observation of the Continuous Phase Transition in Determining the High Thermoelectric Performance of Polycrystalline Sn_0.98_Se. J. Phys. Chem. Lett. 2019, 10, 6512–6517. 10.1021/acs.jpclett.9b02818. 31597419

[ref190] Carlton C. E.; Ferreira P. J. In Situ TEM Nanoindentation of Nanoparticles. Micron 2012, 43, 1134–1139. 10.1016/j.micron.2012.03.002. 22484052

[ref191] Chen H. Y.; Zhao X. B.; Zhu T. J.; Jiang J. Z.; Stiewe C.; Lathe C.; Mueller E. In Situ Energy Dispersive X-Ray Diffraction Study of Iron Disilicide Thermoelectric Materials. J. Phys. Chem. Solids 2008, 69, 2013–2018. 10.1016/j.jpcs.2008.02.012.

[ref192] Guin S. N.; Sanyal D.; Biswas K. The Effect of Order–Disorder Phase Transitions and Band Gap Evolution on the Thermoelectric Properties of AgCuS Nanocrystals. Chem. Sci. 2016, 7, 534–543. 10.1039/C5SC02966J. 29896345 PMC5952868

[ref193] Perez M.; Perrard F.; Massardier-Jourdan V.; Kleber X.; Schmitt V.; Deschamps A. Low Temperature Solubility Limit of Copper in Iron. Mater. Sci. Forum 2005, 500–501, 631–638. 10.4028/www.scientific.net/MSF.500-501.631.

[ref194] Sun Y.; Zuo X.; Sankaranarayanan S. K. R. S.; Peng S.; Narayanan B.; Kamath G. Quantitative 3D Evolution of Colloidal Nanoparticle Oxidation in Solution. Science 2017, 356, 303–307. 10.1126/science.aaf6792. 28428422

[ref195] Li J.-l.; Yin J.-h.; Ji T.; Feng Y.; Liu Y.-y.; Zhao H.; Li Y.-p.; Zhu C.-c.; Yue D.; Su B.; et al. Microstructure Evolution Effect on High-Temperature Thermal Conductivity of LDPE/BNNS Investigated by in-Situ SAXS. Mater. Lett. 2019, 234, 74–78. 10.1016/j.matlet.2018.09.061.

[ref196] Wang J.; Fan W.; Yang J.; Da Z.; Yang X.; Chen K.; Yu H.; Cheng X. Tetragonal–Orthorhombic–Cubic Phase Transitions in Ag_2_Se Nanocrystals. Chem. Mater. 2014, 26, 5647–5653. 10.1021/cm502317g.

[ref197] Chua A. S.; Brochu M.; Bishop D. P. Spark Plasma Sintering of Prealloyed Aluminium Powders. Powder Metall. 2015, 58, 51–60. 10.1179/1743290114Y.0000000105.

[ref198] Liz-Marzán L. M.; Kagan C. R.; Millstone J. E. Reproducibility in Nanocrystal Synthesis? Watch out for Impurities!. ACS Nano 2020, 14, 6359–6361. 10.1021/acsnano.0c04709. 32575172

[ref199] Baranov D.; Lynch M. J.; Curtis A. C.; Carollo A. R.; Douglass C. R.; Mateo-Tejada A. M.; Jonas D. M. Purification of Oleylamine for Materials Synthesis and Spectroscopic Diagnostics for Trans Isomers. Chem. Mater. 2019, 31, 1223–1230. 10.1021/acs.chemmater.8b04198.

[ref200] Lang E. N.; Pintro C. J.; Claridge S. A. Trans and Saturated Alkyl Impurities in Technical-Grade Oleylamine: Limited Miscibility and Impacts on Nanocrystal Growth. Chem. Mater. 2022, 34, 5273–5282. 10.1021/acs.chemmater.2c00945.

[ref201] Park K. T.; Lee T.; Ko Y.; Cho Y. S.; Park C. R.; Kim H. High-Performance Thermoelectric Fabric Based on a Stitched Carbon Nanotube Fiber. ACS Appl. Mater. Interfaces 2021, 13, 6257–6264. 10.1021/acsami.0c20252. 33508940

[ref202] Schlichting K. W.; Padture N. P.; Klemens P. G. Thermal Conductivity of Dense and Porous Yttria-Stabilized Zirconia. J. Mater. Sci. 2001, 36, 3003–3010. 10.1023/A:1017970924312.

[ref203] Adachi J.; Kurosaki K.; Uno M.; Yamanaka S. Effect of Porosity on Thermal and Electrical Properties of Polycrystalline Bulk ZrN Prepared by Spark Plasma Sintering. J. Alloys Compd. 2007, 432, 7–10. 10.1016/j.jallcom.2006.05.115.

[ref204] Ondracek G.; Schulz B. The Porosity Dependence of the Thermal Conductivity for Nuclear Fuels. J. Nucl. Mater. 1973, 46, 253–258. 10.1016/0022-3115(73)90039-1.

[ref205] Lin Y.; Wood M.; Imasato K.; Kuo J. J.; Lam D.; Mortazavi A. N.; Slade T. J.; Hodge S. A.; Xi K.; Kanatzidis M. G.; et al. Expression of Interfacial Seebeck Coefficient through Grain Boundary Engineering with Multi-Layer Graphene Nanoplatelets. Energy Environ. Sci. 2020, 13, 4114–4121. 10.1039/D0EE02490B.

[ref206] Kuo J. J.; Kang S. D.; Imasato K.; Tamaki H.; Ohno S.; Kanno T.; Snyder G. J. Grain Boundary Dominated Charge Transport in Mg_3_Sb_2_-Based Compounds. Energy Environ. Sci. 2018, 11, 429–434. 10.1039/C7EE03326E.

[ref207] Xu B.; Feng T.; Agne M. T.; Zhou L.; Ruan X.; Snyder G. J.; Wu Y. Highly Porous Thermoelectric Nanocomposites with Low Thermal Conductivity and High Figure of Merit from Large-Scale Solution-Synthesized Bi_2_Te_2.5_Se_0.5_ Hollow Nanostructures. Angew. Chemie - Int. Ed. 2017, 56, 3546–3551. 10.1002/anie.201612041. 28079961

[ref208] Xu L.; Yang Y.; Hu Z. W.; Yu S. H. Comparison Study on the Stability of Copper Nanowires and Their Oxidation Kinetics in Gas and Liquid. ACS Nano 2016, 10, 3823–3834. 10.1021/acsnano.6b00704. 26938982

[ref209] Achord J. M.; Hussey C. L. Determination of Dissolved Oxygen in Nonaqueous Electrochemical Solvents. Anal. Chem. 1980, 52, 601–602. 10.1021/ac50053a061.

[ref210] Battino R.; Rettich T. R.; Tominaga T. The Solubility of Oxygen and Ozone in Liquids. J. Phys. Chem. Ref. Data 1983, 12, 163–178. 10.1063/1.555680.

[ref211] Bard A. J.; Faulkner L. R. Electrochemical Methods: Fundamentals and Applications, 2nd ed.; Wiley: New York, NY, 2000.

[ref212] Xu L.; Liang H. W.; Yang Y.; Yu S. H. Stability and Reactivity: Positive and Negative Aspects for Nanoparticle Processing. Chem. Rev. 2018, 118, 3209–3250. 10.1021/acs.chemrev.7b00208. 29517229

[ref213] Bhanushali S.; Ghosh P.; Ganesh A.; Cheng W. 1D Copper Nanostructures: Progress, Challenges and Opportunities. Small 2015, 11, 1232–1252. 10.1002/smll.201402295. 25504816

[ref214] Masitas R. A.; Zamborini F. P. Oxidation of Highly Unstable < 4 Nm Diameter Gold Nanoparticles 850 MV Negative of the Bulk Oxidation Potential. J. Am. Chem. Soc. 2012, 134, 5014–5017. 10.1021/ja2108933. 22372940

[ref215] Mikhlin Y. L.; Vishnyakova E. A.; Romanchenko A. S.; Saikova S. V.; Likhatski M. N.; Larichev Y. V.; Tuzikov F. V.; Zaikovskii V. I.; Zharkov S. M. Oxidation of Ag Nanoparticles in Aqueous Media: Effect of Particle Size and Capping. Appl. Surf. Sci. 2014, 297, 75–83. 10.1016/j.apsusc.2014.01.081.

[ref216] De Kergommeaux A.; Faure-Vincent J.; Pron A.; De Bettignies R.; Malaman B.; Reiss P. Surface Oxidation of Tin Chalcogenide Nanocrystals Revealed by ^119^Sn-Mössbauer Spectroscopy. J. Am. Chem. Soc. 2012, 134, 11659–11666. 10.1021/ja3033313. 22691030

[ref217] Petermann N.; Stötzel J.; Stein N.; Kessler V.; Wiggers H.; Theissmann R.; Schierning G.; Schmechel R. Thermoelectrics from Silicon Nanoparticles: The Influence of Native Oxide. Eur. Phys. J. B 2015, 88, 163 10.1140/epjb/e2015-50594-7.

[ref218] Hines D. A.; Kamat P. V. Recent Advances in Quantum Dot Surface Chemistry. ACS Appl. Mater. Interfaces 2014, 6, 3041–3057. 10.1021/am405196u. 24506801

[ref219] Sykora M.; Koposov A. Y.; McGuire J. A.; Schulze R. K.; Tretiak O.; Pietryga J. M.; Klimov V. I. Effect of Air Exposure on Surface Properties, Electronic Structure, and Carrier Relaxation in PbSe Nanocrystals. ACS Nano 2010, 4, 2021–2034. 10.1021/nn100131w. 20369900

[ref220] Müller J.; Lupton J. M.; Rogach A. L.; Feldmann J.; Talapin D. V.; Weller H. Air-Induced Fluorescence Bursts from Single Semiconductor Nanocrystals. Appl. Phys. Lett. 2004, 85, 381 10.1063/1.1769585.

[ref221] Shu G.-W.; Lee W.-Z.; Shu I.-J.; Shen J.-L.; Lin J. C.-A.; Chang W. H.; Ruaan R.-C.; Chou W. C. Photoluminescence of Colloidal CdSe/ZnS Quantum Dots Under Oxygen Atmosphere. IEEE Trans. Nanotechnol. 2005, 4, 632–636. 10.1109/TNANO.2005.851445.

[ref222] Lee Y. K.; Luo Z.; Cho S. P.; Kanatzidis M. G.; Chung I. Surface Oxide Removal for Polycrystalline SnSe Reveals Near-Single-Crystal Thermoelectric Performance. Joule 2019, 3, 719–731. 10.1016/j.joule.2019.01.001.

[ref223] Jørgensen L. R.; Iversen B. B. Characterizing Thermoelectric Stability. Dalt. Trans. 2022, 51, 3807–3816. 10.1039/D1DT04001D. 35147624

[ref224] Zeuthen C. M.; Thorup P. S.; Roth N.; Iversen B. B. Reconciling Crystallographic and Physical Property Measurements on Thermoelectric Lead Sulfide. J. Am. Chem. Soc. 2019, 141, 8146–8157. 10.1021/jacs.9b00043. 31042374

[ref225] Sharma P. K.; Senguttuvan T. D.; Sharma V. K.; Patro P.; Chaudhary S. Effect of Bismuth Doping and SiC Nanodispersion on the Thermoelectric Properties of Solution-Processed PbTe. J. Alloys Compd. 2022, 915, 165390 10.1016/j.jallcom.2022.165390.

[ref226] Pei Y.; Lalonde A.; Iwanaga S.; Snyder G. J. High Thermoelectric Figure of Merit in Heavy Hole Dominated PbTe. Energy Environ. Sci. 2011, 4, 2085–2089. 10.1039/c0ee00456a.

[ref227] Pei Y.; Heinz N. A.; Snyder G. J. Alloying to Increase the Band Gap for Improving Thermoelectric Properties of Ag_2_Te. J. Mater. Chem. 2011, 21, 18256–18260. 10.1039/c1jm13888j.

[ref228] Yang C. Y.; Ding Y. F.; Huang D.; Wang J.; Yao Z. F.; Huang C. X.; Lu Y.; Un H. I.; Zhuang F. D.; Dou J. H.; et al. A Thermally Activated and Highly Miscible Dopant for N-Type Organic Thermoelectrics. Nat. Commun. 2020, 11, 3292 10.1038/s41467-020-17063-1. 32620924 PMC7335177

[ref229] Zhong F.; Yin X.; Chen Z.; Gao C.; Wang L. Significantly Reduced Thermal-Activation Energy for Hole Transport via Simple Donor Engineering: Understanding the Role of Molecular Parameters for Thermoelectric Behaviors. ACS Appl. Mater. Interfaces 2020, 12, 26276–26285. 10.1021/acsami.0c05771. 32421324

[ref230] James D.; Lu X.; Nguyen A. C.; Morelli D.; Brock S. L. Design of Lead Telluride Based Thermoelectric Materials through Incorporation of Lead Sulfide Inclusions or Ligand Stripping of Nanosized Building Block. J. Phys. Chem. C 2015, 119, 4635–4644. 10.1021/jp5127046.

[ref231] Hu C.; Xia K.; Fu C.; Zhao X.; Zhu T. Carrier Grain Boundary Scattering in Thermoelectric Materials. Energy Environ. Sci. 2022, 15, 1406–1422. 10.1039/D1EE03802H.

[ref232] Imasato K.; Fu C.; Pan Y.; Wood M.; Kuo J. J.; Felser C.; Snyder G. J. Metallic N-Type Mg_3_Sb_2_ Single Crystals Demonstrate the Absence of Ionized Impurity Scattering and Enhanced Thermoelectric Performance. Adv. Mater. 2020, 32, 1908218 10.1002/adma.201908218. 32115799

[ref233] Niu Z.; Li Y. Removal and Utilization of Capping Agents in Nanocatalysis. Chem. Mater. 2014, 26, 72–83. 10.1021/cm4022479.

[ref234] Doris S. E.; Lynch J. J.; Li C.; Wills A. W.; Urban J. J.; Helms B. A. Mechanistic Insight into the Formation of Cationic Naked Nanocrystals Generated under Equilibrium Control. J. Am. Chem. Soc. 2014, 136, 15702–15710. 10.1021/ja508675t. 25302526

